# An addressable packing parameter approach for reversibly tuning the assembly of oligo(aniline)-based supra-amphiphiles†
†Electronic supplementary information (ESI) available. See DOI: 10.1039/c8sc00068a


**DOI:** 10.1039/c8sc00068a

**Published:** 2018-04-02

**Authors:** Wei Lyu, Maha Alotaibi, O. Alexander Bell, Kazuyoshi Watanabe, Robert Harniman, Benjamin M. Mills, Annela M. Seddon, Sarah E. Rogers, Stephen M. King, Wei Yan, Charl F. J. Faul

**Affiliations:** a School of Chemistry , University of Bristol , Bristol , BS8 1TS , UK . Email: charl.faul@bristol.ac.uk; b Department of Environmental Science and Engineering , Xi'an Jiaotong University , 710049 , Xi'an , P. R. China; c Chemistry Department , Faculty of Science , King Abdul Aziz University , Jeddah , Kingdom of Saudi Arabia; d School of Physics , H. H. Wills Physics Laboratory , University of Bristol , Tyndall Avenue , Bristol , BS8 1FD , UK; e ISIS Pulsed Neutron & Muon Source , STFC Rutherford Appleton Laboratory , Harwell Campus , Didcot , OX11 0QX , UK; f Bristol Centre for Functional Nanomaterials , H. H. Wills Physics Laboratory , University of Bristol , Tyndall Avenue , Bristol , BS8 1FD , UK

## Abstract

We present a newly developed approach to non-covalently address the packing parameter of an electroactive amphiphile. The pH-responsive reversible switching of a tetra(aniline)-based cationic amphiphile, **TANI**-pentyl trimethylammonium bromide (**TANI-PTAB**), between self-assembled vesicles and nanowires by acid/base chemistry in aqueous solution is used to exemplify this approach. Trifluoroacetic acid (**TFA**) was selected as a prototypical acid to form emeraldine salt (ES) state (**TANI(TFA)_2_-PTAB**) vesicles for this new class of small-molecule supramolecular amphiphiles. UV-vis-NIR spectroscopy, transmission electron microscopy (TEM), tapping-mode atomic force microscopy (AFM), and fluorescence spectroscopy were used to investigate the reversible structural transformation from vesicles to nanowires. We show that utilising different protonic acid-dopants for **TANI-PTAB** can regulate the packing parameter, and thus the final self-assembled structures, in a predictable fashion. We envisage potential application of this concept as smart and switchable delivery systems.

## Abstract 2

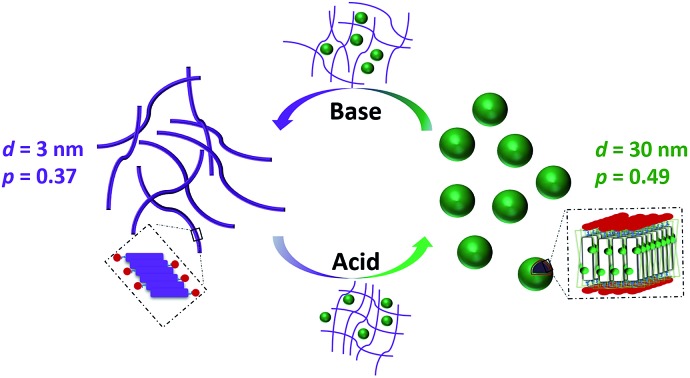
An addressable packing parameter approach was developed for reversibly tuning the self-assembly of oligo(aniline)-based supra-amphiphiles.

## Introduction

The formation of well-defined self-assembled supramolecular nanostructures continues to attract considerable attention as a strategy to prepare active and addressable materials for a wide range of applications and devices.^1^,^2^ Amphiphiles, a well-known class of materials whose self-assembly behaviour has been thoroughly studied in aqueous environments, are of interest because of their applications in the consumer products, personal care, and pharmaceutical industries.^3^ Their development has included the study and application of polymeric surfactants,^4^ and novel sugar-based and CO_2_-philic amphiphiles^5^ for sustainable and green chemistry applications.

A recent development in the area of novel amphiphiles has been the advent of the so-called supra-amphiphiles, a new class of supramolecular amphiphilic constructs. To produce such constructs, carefully designed sub-units produced using covalent chemistry are allowed to interact *via* various non-covalent interactions to form new amphiphiles. Such non-covalent interactions include hydrogen-bonding, host-guest, π-stacking, charge-transfer and electrostatic interactions.^6^–^8^ Once formed, these supra-amphiphiles can assemble, just like classical amphiphiles, into micelles, vesicles, or nanofibers through the careful choice and tailoring of further non-covalent interactions, or even dynamic covalent bonds.^9^ Such control enables the design of dynamic and smart materials with the capability to respond to external stimuli, such as photo-irradiation,^10^ changes in temperature,^11^ pH,^12^ redox potential,^13^ solvent type,^14^ and concentration,^15^ and form the basis for investigations into the production of novel tuneable materials.

Oligo(aniline)s are a class of conjugated oligomeric materials that act as model compounds of the well-known conducting polymer poly(aniline) (**PANI**). These conjugated materials have attracted increasing academic interest as conjugated cores for the construction of new functional materials, amphiphilic copolymers, and surfactants.^16^–^21^ Compared with **PANI**, oligo(aniline)s possess well-defined and monodisperse molecular structures, and exhibit excellent solubility and processability. These features have enabled the design of oligo(aniline)-based materials with well-defined functionalities, as well as new properties and applications that are unavailable to **PANI**.^22^–^26^ Among oligo(aniline)s, tetra(aniline) (**TANI**) is the shortest oligomer that can exhibit the full range of oxidation states,^22^ including the emeraldine base (EB) state, which is required for pH-responsive switching. The unique pH- and redox-switchable properties of oligo(aniline)s endow amphiphiles based on this motif with stimuli-responsive self-assembly characteristics.^16^,^17^ To date, several reports exist of oligo(aniline)-containing amphiphiles that show redox-induced structural changes. Park and co-workers reported **TANI**-*b*-poly(ethylene glycol), which showed reversible electrochemical switching between self-assembled vesicles and micelles.^16^ They recently reported redox-responsive aggregation–disaggregation behavior between the EB and the leucoemeraldine base state (LEB) of a diaminohepta(aniline)-based multi-block rod-coil structure.^27^ Wu *et al.* obtained electrochemically switchable vesicles from a **TANI**-*b*-poly(*N*-isopropyl acrylamide) polymer. Application of an oxidizing voltage at 0.6 V led to the destruction of these vesicles, reportedly by the removal of the structure-directing amine–imine hydrogen bonding.^17^ These reports are, to the best of our knowledge, the only previous studies of redox-responsive self-assembly of oligo(aniline) amphiphiles.

Compared with traditional pH-responsive systems, the unique pH-switchable properties of oligo(aniline)s endow an additional means of control in the form of **TANI**'s well-known switchable conductivity,^22^ as well as the more recently explored switchable optical properties (specifically refractive indices).^28^ It was therefore surprising to find that despite the unique doping properties of aniline-based materials, reports of pH-induced structural changes (*i.e.*, acid doping and base de-doping) directly affecting the self-assembly of oligo(aniline)s (both amphiphilic and non-amphiphilic) are rare. Previous work has relied solely on the structural differences produced by switching between the fully reduced LEB and the half-oxidised EB states of **TANI**, while the doped and conducting emeraldine salt (ES) state has been largely overlooked. Consequently, we sought to further develop a general strategy to design oligo(aniline)-based pH-responsive smart systems.

One aspect of the unique ability of oligo(aniline)-based materials to be doped that has not been exploited is the use of dopants to address not only the redox state, but also the volume of the electroactive component (induced volume changes in **PANI** have been exploited widely for the production of soft actuators^29^). In the case of classical amphiphiles, such changes in molecular volume (*v*) will lead to changes of the well-known surfactant packing parameter *p*. The dimensionless packing parameter, *p*, is widely used to link the structure of an amphiphile to predicted micelle morphology in aqueous solutions.1
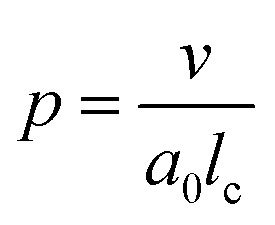



As defined in eqn (1), *v* is the volume of the hydrophobic section, *a*_0_ is the optimal headgroup area per amphiphile and *l*_c_ is the (critical) hydrophobic tail length.^30^ The packing parameter, *p*, predicts that spherical micelles are favoured when 
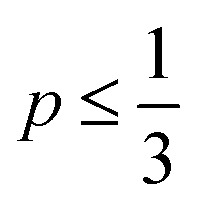
, cylindrical micelles are formed at 
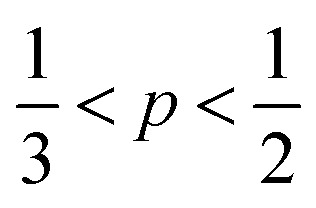
 , flexible bilayers and vesicles arise when 
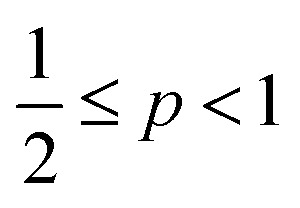
, planar bilayers are favoured at *p* = 1 and inverted micelles appear when *p* > 1.^31^

Changes in *p* are generally limited to tuning the headgroup area for classical covalent amphiphiles (through changes in concentration or counterions). For polymeric supra-amphiphiles this approach (*i.e.*, changes in *p*) has led to reversible switching of the assembled nanostructures.^32^–^34^

**Scheme 1:**
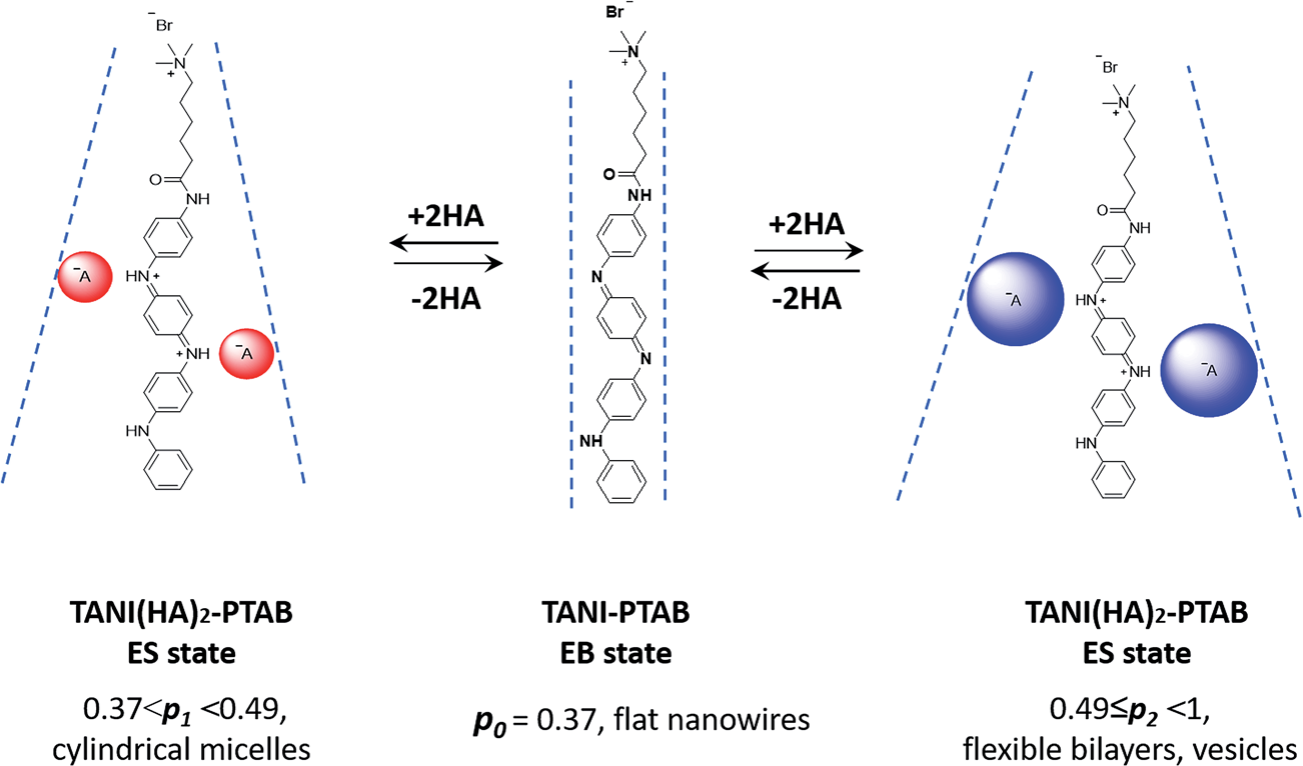
Schematic showing the unique reversible doping/de-doping properties of the **TANI**-based electroactive amphiphile **TANI-PTAB**. Tail volume, and thus the packing parameter, can be changed through the use of different dopants.

It is therefore envisaged that this strategy will also be suitable for amphiphiles based on our **TANI** motif: the electroactive tail of a **TANI**-based amphiphile will be available for reversible protonic acid doping and de-doping, and thus lead to reversibly tuneable changes in *v* and *p* and, consequently, the formed self-assembled structures; *i.e.*, a reversibly addressable low molecular weight electrostatic supra-amphiphile (eSA).^35^ In addition, changing the type (and thus volume) of the dopant would provide a simple strategy to tune packing parameters and thus formed supramolecular assembled structures in a facile fashion, as shown in Scheme 1. We term this approach the “addressable packing parameter (APP)” approach.

We recently reported the aqueous self-assembly of a **TANI**-based cationic amphiphile, **TANI-PTAB** with a packing parameter *p* = 0.37, see Scheme 1, into 3 nm-wide flat nanowires or nanobelts. This assembly was driven primarily by the hydrophobic effect, with structure-directing hydrogen bonding and π–π stacking interactions occurring between **TANI** molecules.^19^

Here, based on our previous work and the expected influence of noncovalent dopants on the volume of the **TANI**-based surfactant tail and thus the packing parameter, we explore an unprecedented method of tuning the self-assembly of small-molecule amphiphiles. Starting with **TANI-PTAB** in the EB state, *p* = 0.37, trifluoroacetic acid (**TFA**) was selected as an initial model acid to form the doped ES-state amphiphile, **TANI(TFA)_2_-PTAB**, with a calculated packing parameter *p* = 0.49. We demonstrate reversible switching of this system between nanowires (*p* = 0.37) and vesicles (*p* = 0.49), in response to changes in pH that control the reversible protonic doping of the **TANI**-based surfactant tail. Moreover, we show the generality of this approach by using dopants of different sizes (and volumes) to change the packing parameter *p*, leading to the reversible tuning of the self-assembled amphiphile structures, *e.g.*, from wires to worm-like micelles and bilayer structures and back to wires.

## Experimental section

### Synthesis

**TANI-PTAB** was synthesized according to the previously reported route.^19^ Full synthetic details and characterization are available in the ESI.†

### Theoretical estimation of packing parameter of EB **TANI-PTAB** and ES **TANI(HX)_2_-PTAB**

The calculated packing parameters of EB **TANI-PTAB** and ES **TANI(HX)_2_-PTAB** (HX = protonic acid dopant) species were estimated according to eqn (1). The parameters *v* and *l*_c_ were estimated by QSAR calculations based on DFT models.^36^ For **TANI**-based amphiphiles bearing an alkyltrimethylammonium bromide headgroup, the cross-sectional area of the polar group, *a*_0_, was estimated to be 0.54 nm^2^ by considering the bond lengths and bond angles, as in previous reports.^37^ Full computational details and results are shown in the ESI.†

### Doping studies

An aqueous solution of **TFA** (2 eq., 32 mM, 125 μL) was added to EB **TANI-PTAB** solid (1 eq., 1.2 mg, 2 mmol) to achieve a doping ratio of 2 **TFA** : 1 **TANI-PTAB**, followed by dilution with the required volume of deionised water to achieve the desired final concentration. The solutions were shaken and sonicated for 5 min to ensure homogeneity, and then left undisturbed overnight (RT) before analysis. Doped samples were stored in the fridge between experiments.

### Characterization

UV-vis-NIR spectra were recorded using a Shimadzu UV2600 spectrophotometer. FT-IR spectra were collected using a Perkin Elmer Spectrum 100 FT-IR spectrometer with attenuated total reflectance cell; samples were freeze-dried before measurements. Transmission electron microscopy (TEM) was performed on a JEOL 1400 TEM with a tungsten filament, operated at a frequency of 120 kV and equipped with a 4 Mp Gatan Orius 830 digital camera using the Gatan DigitalMicrograph software. Samples (5 μL) were drop-cast onto carbon-coated copper grids and allowed to rest for 60 s, before the excess solution was wicked away. Samples were stained with a 1% aqueous uranyl acetate solution for 30 s. Dynamic light scattering (DLS) was carried out on a Malvern Zetasize Nano-S apparatus with a 532 nm laser. Atomic force microscopy (AFM) was performed using a Bruker Multimode with a Nanoscope V controller and Picoforce Extender. Samples were drop-cast onto either carbon-coated copper grids or freshly cleaved mica. Fluorescence spectra were obtained using a Varian Cary Eclipse Fluorimeter. Samples for fluorescence analysis were prepared by adding a solution of pyrene in acetone (20 μL, 0.5 mg mL^–1^) to aqueous ES **TANI(TFA)_2_-PTAB** (2 mL) solutions of varying concentrations, followed by purging with argon. A pyrene emission spectrum (between 360 nm and 600 nm, excitation at 337 nm) was recorded for each solution. Please see ESI Page S18† for additional comments on attempts to determine the critical aggregation concentration (CAC).

X-ray scattering data were collected on a Ganesha SAXS/WAXS instrument (SAXSLAB) at room temperature over a Q-range of 0.001 < *Q* < 0.7 Å^–1^ where2
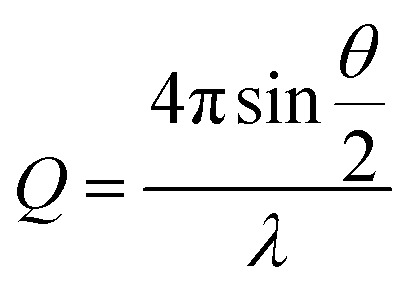



*Q* is the scattering vector, *θ* is the scattering angle and *λ* is the incident wavelength. The source is a GeniX3D source (made by Xenocs) with a wavelength of 1.5406 Å. The detector is a Pilatus 300 K by Dectris. Samples were loaded into borosilicate glass capillaries (Capillary Tube Supplies) and sealed with UV-curable adhesive (Norland Optical Adhesive). Data were collected over an exposure time of 3 hours and background-corrected with TFA, and intensity measurements were corrected for tube thickness. Data analysis was performed using SasView (versions 3.0x).^38^

Small-angle neutron scattering (SANS) data were obtained on the LOQ small-angle diffractometer at the ISIS Pulsed Neutron Source (STFC Rutherford Appleton Laboratory, Didcot, U.K.).^39^,^40^ This is a fixed-geometry “white beam” time-of-flight instrument, which utilizes neutrons with wavelengths between 2.2 and 10 Å. Data are simultaneously recorded on two, two-dimensional, position-sensitive, neutron detectors, to provide a simultaneous *Q* range of 0.008–1.6 Å^–1^. Each sample and background was placed in 2 mm path length quartz cuvettes and was measured for a total of 5 hours to gather data of high statistical precision. The beam diameter was 8 mm. Each raw scattering data set was then corrected for the detector efficiencies, sample transmission and background scattering and converted to scattering cross-section data (∂*Σ*/∂*Ω vs. Q*) using the instrument-specific software.^41^ These data were placed on an absolute scale (cm^–1^) using the scattering from a standard sample (a solid blend of hydrogenous and perdeuterated polystyrene) in accordance with established procedures.^42^ SANS data were subsequently collected, over the much lower *Q*-range of 0.0015–0.25 Å^–1^, on the SANS2D small-angle diffractometer at ISIS, utilizing an incident wavelength range of 1.75–12.5 Å and employing an instrument configuration of *L*1 = *L*2 = 12 m (where *L*1 = source-sample distance, and *L*2 = sample-detector distance), with the 1 m^2^ main detector offset vertically by 60 mm and sideways by 100 mm.^39^,^43^ The beam diameter was again 8 mm. These data were processed in an analogous manner to that from LOQ. The SANS data were also analysed using SasView (versions 3.0x).^38^

## Results and discussion

### Self-assembly of ES **TANI(TFA)_2_-PTAB**

In our previously reported studies into the self-assembly of **TANI-PTAB**,^19^ we focused mainly on the undoped EB state, forming infinitely long 3 nm wide π-stacked nanowires in solution. Modelling of solution SAXS data indicated that these structures could be described as flat and tape-like, with widths of 3 nm. Initial doping studies with CSA, the prototypical **PANI** acid dopant,^19^ showed the formation of thicker (6 nm) less well-defined conductive wire-like constructs. Simple calculations (see Scheme 1 and Table 1) suggested that *p* changed from 0.37 (for EB **TANI-PTAB**) to 0.73 for ES **TANI(CSA)_2_-PTAB** on the addition of CSA, but these changes and their potential influence on assembly was not further explored in any way.

**Table 1:** Estimated parameter and expected self-assembled structure for **TANI-PTAB** systems

Molecule	p*K*_a_	*V*(Å^3^)	*l*_c_(Å)	*a*_0_(Å^2^)	*p*	Expected self-assembled structure
**TANI-PTAB**	—	474.10	23.91	54.00	0.37	Cylindrical micelles
**TANI(HCl)_2_-PTAB**	–7.00	535.31	24.28	54.00	0.41	Cylindrical micelles
**TANI(HNO_3_)_2_-PTAB**	–1.40	569.71	24.01	54.00	0.44	Cylindrical micelles
**TANI(AcOH)_2_-PTAB**	4.76	607.62	24.04	54.00	0.47	Cylindrical micelles
**TANI(TFA)_2_-PTAB**	0.23	634.87	24.03	54.00	0.49	Flexible bilayers, vesicles
**TANI(DCA)_2_-PTAB**	1.35	663.39	23.94	54.00	0.51	Flexible bilayers, vesicles
**TANI(CSA)_2_-PTAB**	1.20	933.91	23.78	54.00	0.73	Flexible bilayers, vesicles
**TANI(BinPO_4_H)_2_-PTAB**	3.37	1160.28	24.06	54.00	0.89	Flexible bilayers, vesicles

Careful examination and further investigations into a range of acid dopants, to explore this proposed switchability, led us to **TFA** as the dopant of choice for the following reasons: (a) **TFA** possessed the capacity to dope **TANI**-based materials with a p*K*_a_ = 0.23; (b) the calculated value of *p* after doping, *i.e.* for the doped ES **TANI(TFA)_2_-PTAB**, is = 0.49; and (c) a series of further related acids with different volumes would be available for additional investigations.

**Fig. 1:**
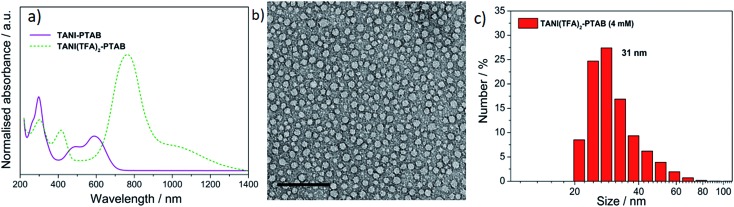
(a) Normalized UV-vis-NIR spectra of EB **TANI-PTAB** and **TFA**-doped ES **TANI(TFA)_2_-PTAB** samples. (b) TEM image of a 4 mM **TANI(TFA)_2_-PTAB** sample (stained with 1% uranyl acetate). (c) Number distribution of object sizes by DLS for a 4 mM **TANI(TFA)_2_-PTAB** solution. Scale bar: 200 nm.

As expected, doping EB **TANI-PTAB** with **TFA** has a profound effect on the formed self-assembled structures: the addition of **TFA** converts EB **TANI-PTAB** with its well-defined 3 nm-wide nanowire morphology to doped ES **TANI(TFA)_2_-PTAB**, now with a very well-defined vesicular structure. The capacity of **TFA** to dope EB **TANI-PTAB** to the ES state was confirmed by UV-vis-NIR absorption spectroscopy.^44^ As shown in Fig. 1a, three maxima were observed in the UV-vis-NIR spectrum of **TANI(TFA)_2_-PTAB** at 1 mM in water, located at 302 nm, 420 nm, and 780 nm. These maxima originate from π-electron transitions in the **TANI-PTAB** molecule that are comparable to the π–π*, polaron-π*, and π-polaron band transitions in PANI-ES, respectively,^22^,^45^ and are characteristic of the ES state of doped oligo(aniline) species. Recent^46^ TD-DFT calculations on the core **TANI** electroactive unit have shown that absorption maxima in the range 780–800 nm correspond to the presence of the triplet ES state. The presence of these bands, as well as the absence of the absorption features typical for EB-state materials (especially the band at 600 nm) confirm that fully doped ES **TANI(TFA)_2_-PTAB** was formed. These conclusions were further confirmed by FT-IR spectra of **TANI-PTAB** and 2 mM **TANI(TFA)_2_-PTAB**, recorded in the solid state (Fig. S7†). Circular objects with average diameters of 27 ± 2 nm were observed by conventional TEM (Fig. 1b, S9a†), and DLS was used to confirm the existence of objects with similar size in solution. The average hydrodynamic diameter was determined to be 31 ± 7 nm (Fig. 1c), which agrees very well with the measurements from TEM, since the hydrodynamic diameter is expected to be larger than that of the dried sample.^47^

**Fig. 2:**
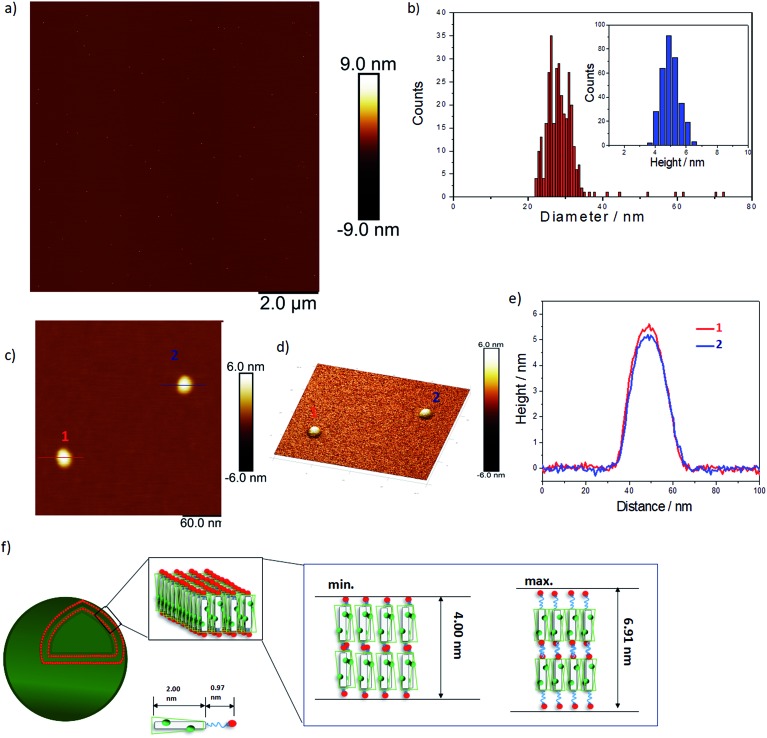
(a) AFM image of 2 mM **TANI(TFA)_2_-PTAB** sample on a mica surface. (b) The corresponding diameter and height distributions counted from 317 particles. (c) AFM image of two typical vesicles, (d) the corresponding 3D image with the *Z*-ratio increased by a factor of 3 for clarity and (e) their cross-sectional height profile. (f) A proposed scheme showing the possible molecular packing model for the self-assembled vesicles.

We used AFM to further investigate the topology of these objects. AFM images of a **TANI(TFA)_2_-PTAB** solution (2 mM), drop-cast on a mica surface, are shown in Fig. 2. Highly monodisperse circular structures were observed on the surface (Fig. 2a, also embedded in films – see Fig. S8†), indicating that these objects were not droplets formed by drying. The average diameter and height of these objects, calculated from 317 counted objects, were 28.80 ± 3.33 nm and 5.01 ± 0.57 nm, respectively (Fig. 2b). The value of the average diameter was found to be in excellent agreement with those measured by both DLS and TEM. Two representative objects were selected to show their detail in higher resolution and 3D images (Fig. 2c, 2d, respectively). The measured heights of these two objects were 5.5 nm and 5 nm, respectively (Fig. 2e). Given the theoretical stretched length of an upright **TANI(TFA)_2_-PTAB** molecule (2.97 nm, of which the rigid **TANI** block makes up 2 nm with 0.97 nm of flexible alkyl chains), we deduce that the height is determined by the thickness of an interdigitated double layer of **TANI(TFA)_2_-PTAB**. This layer is calculated to have a minimum height of ∼4 nm (with fully interdigitated and overlapping π-conjugated surfaces and no tilt with respect to the surface) and maximum height of ∼6.9 nm (an upright, non-interdigitated state), as shown in the proposed molecular packing models in Fig. 2f. Moreover, the absence of the characteristic adsorption of strong asymmetric inter-chain NH^+^···N hydrogen bonds in the FT-IR spectrum of **TANI(TFA)_2_-PTAB**, shown in Fig. S7,† support the proposed packing model. Therefore, combining the measured average diameter with the proposed interdigitated double-layer packing model, we deduced that these objects are hollow double-layer vesicles.

SAXS and SANS investigations (in H_2_O- and D_2_O-based media, respectively) were also used to probe the shape and size of the objects formed in the 4 mM **TANI(TFA)_2_-PTAB** system; see the ESI† for representative data. The SANS data possessed superior scattering contrast, but both sets of measurements showed a pronounced ∼*Q*^–2^ dependence. This behaviour is consistent with *either* the overall scattering from discoidal particles/platelets, *or* the sheet-like scattering from a unilamellar structure such as might surround a vesicle, but unfortunately there is no way to unambiguously differentiate between the two. However, if the TEM and DLS size data are to be believed, and the objects were discs/platelets, then one would expect to see a levelling off in the low-*Q* scattering in the measurement window of the SANS instruments; this behaviour was not observed. Instead, the SANS data start to increase more steeply than *Q*^–2^ below *Q* ∼ 0.004 Å^–1^, equivalent to a length scale of ∼160 nm. The explanation for this observation is unclear. The absence of any oscillations in the SANS data at high-*Q* is likely due to the significant polydispersity of the system (see Fig. 1c), but the absence of a pronounced diffraction peak at high-*Q* also rules out any multilamellar structures.

### Reversible pH-responsive self-assembly

**Fig. 3:**
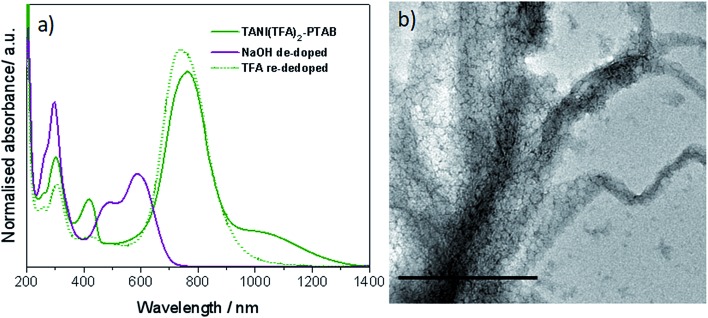
(a) UV-vis-NIR spectra of **TANI(TFA)_2_-PTAB** sample, NaOH de-doped sample and TFA re-doped sample. (b) Control with the addition of NaCl to ES **TANI(TFA)_2_-PTAB**. Scale bar: 500 nm.

Confirmation was sought that the observed changes in packing parameter, and thus self-assembly behaviour, are reversible and driven by an acid-dopant-induced packing parameter change of the addressable amphiphile, rather than a simple change in volume or concentration.^48^ Our starting point was thus to first prepare the ES **TANI(TFA)_2_-PTAB** vesicular structures by doping an EB **TANI-PTAB** fibre-containing solution with **TFA**, as discussed above (Fig. 1b). A small amount of concentrated NaOH solution (0.1 M, 0.03 mL) was then added to **TANI(TFA)_2_-PTAB** (1 mM,^49^ 1.5 mL) to de-dope and alter the pH with minimal change in volume. We observed the full conversion of ES **TANI(TFA)_2_-PTAB** to EB **TANI-PTAB**, as shown in the UV-vis-NIR spectra in Fig. 3a, with the pH changing from 3.0 to 6.2. In a control experiment designed to evaluate the influence of the ionic strength on structure formation, we added identical amounts of NaCl to doped 1 mM **TANI(TFA)_2_-PTAB**. Addition of NaCl produced aggregated vesicles with an average diameter of 29 ± 2 nm (Fig. 3b, S9b†), with no perceptible change in morphology.

**Fig. 4:**
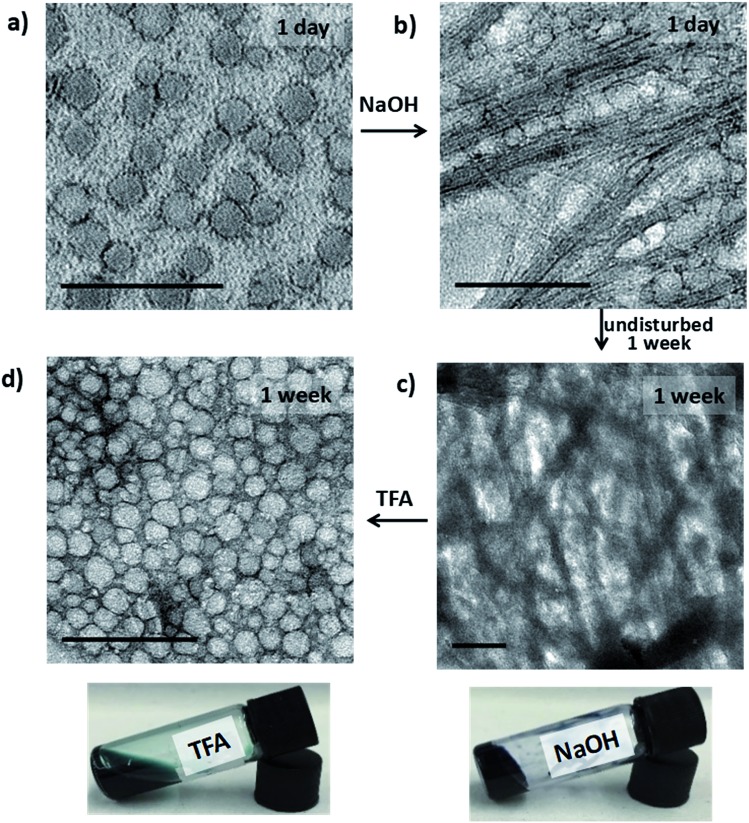
TEM images (stained with 1% uranyl acetate) of (a) **TANI(TFA)_2_-PTAB** (1 mM) after standing 1 day, (b) NaOH-de-doped **TANI-PTAB** after 1 day, (c) the same sample as (b) after 1 week, and (d) **TANI(TFA)_2_-PTAB** (0.92 mM) obtained by treating the same sample as (b, c) with **TFA**, after standing 1 week. Inset photographs (c and d) of the samples showing the gel–sol transition. Scale bars: 200 nm.

Investigation of the structures formed during the following series of doping/de-doping cycles (see Fig. 4) showed the initial co-existence of vesicles and nanowires after addition of NaOH (Fig. 4b). We propose that these structures are indicative of a kinetically trapped state, rather than a thermodynamic minimum, as the UV-vis-NIR data showed that all **TANI** moieties were fully de-doped. This suggestion was confirmed by leaving the sample undisturbed for 1 week to equilibrate, after which a weak gel with a high concentration of bundles of nanowires, visible by TEM (Fig. 4c), formed. We added **TFA** to the fibre-containing gelled samples to further explore the usefulness of the switchable nature of our oligo(aniline) functional unit for structural changes. After leaving the samples to stand undisturbed for a further week, we obtained a low-viscosity solution containing vesicles again, as clearly shown in Fig. 4d (and the inset photos of the gel and solution, respectively). This finding indicates that a fully reversible doping/de-doping-induced morphology switching system was achieved. The average diameter of the obtained vesicular structures was 42 ± 2 nm, although this increase in size (*vs.* initial vesicles) could be attributed to the change in ionic strength.

**Scheme 2:**
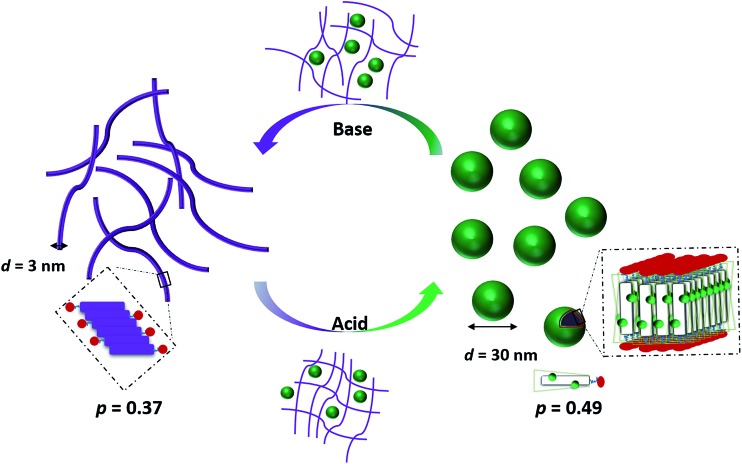
Schematic showing the proposed doping/de-doping responsive self-assembly behaviour of **TANI(TFA)_2_-PTAB** in aqueous solution.

As shown in Scheme 2, the removal of the **TFA** counterions and protons (de-doping) modifies the packing parameter by changing the volume of the **TANI**-containing amphiphile, causing the vesicles formed by the ES state to revert to nanowires.

### Non-covalent packing parameter tuning: generalising the approach

Upon doping with acid, the volume of the hydrophobic section of **TANI** increased, which was reflected in an increased surfactant packing parameter. However, a number of factors (including the size, polarity and chemical nature of the conjugate base) could affect the range of non-covalent interactions among **TANI** molecules, making the doping-induced self-assembly of TANI-based **eSAs** a complex process. In an attempt to gain insight and to start to explore simple variations, dopants of different size and polarity were carefully selected to vary the estimated packing parameter from 0.37 (EB **TANI-PTAB**) through to 0.89 (ES **TANI(HX)_2_-PTAB**), thus providing an exciting opportunity to reversibly tune *p* for our low molecular weight eSA. Please see Table 1 for an overview of the chosen acids, as well as a summary of the relevant molecular parameters.

**Fig. 5:**
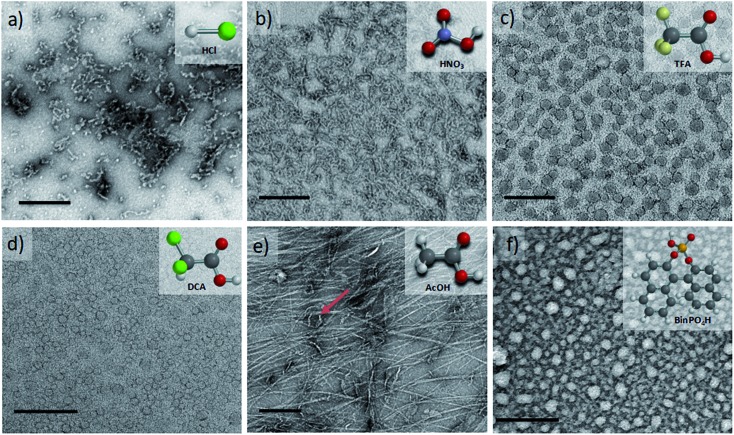
TEM images (1 mM solutions, stained with 1% uranyl acetate) of (a) **TANI(HCl)_2_-PTAB**, (b) **TANI(HNO_3_)_2_-PTAB**, (c) **TANI(TFA)_2_-PTAB**, (d) **TANI(DCA)_2_-PTAB**, (e) **TANI(AcOH)_2_-PTAB** and (f) **TANI(BINPO_4_H)_2_-PTAB**, with corresponding dopant molecular structures inset. Scale bars: 200 nm.

We started our packing parameter investigations with the small inorganic acid dopants HCl and HNO_3_, where *p* was calculated to be 0.41 and 0.44, respectively. Slightly elongated worm-like cylindrical micelles were observed for both **TANI(HCl)_2_-PTAB** (Fig. 5a) and **TANI(HNO_3_)_2_-PTAB** (Fig. 5b), which agreed with the morphology suggested by the packing parameters in Table 1.^50^

Acetic acid (**AcOH**, *p* = 0.47) and dichloroacetic acid (**DCA**, *p* = 0.51) were used as dopants that are analogous to **TFA** and whose packing parameters bracket the value calculated for TFA (*p* = 0.49). We found a significant difference in the formed self-assembled structures: **AcOH** was unable to fully dope **TANI-PTAB** (with a molar ratio of 2 : 1) because of its high p*K*_a_ value (4.76). Partial doping was shown by the presence of peaks attributed to both the EB and ES-state absorption bands in the UV-vis spectra (Fig. S10†), indicating a mixture of the two species. TEM investigations showed that **TANI(AcOH)_2_-PTAB** formed a mixture of nanowires, vesicles and some intermediate states, as shown in Fig. 5e (red arrow). It is clear that the low level of doping (a consequence of the high p*K*_a_) led to an undesired mixture of self-assembled structures, and is therefore not a true reflection of the influence of the dopant on the surfactant tail volume. Increasing the amount of **AcOH** to a mole ratio of 4 : 1 **AcOH : TANI-PTAB** yielded doped vesicular **TANI(AcOH)_2_-PTAB**. However, the simultaneous presence of a small feature at 580 nm in the UV-vis-NIR spectrum, typical of undoped materials, was congruent with the observation by TEM that nanowires remained in the system (Fig. S11a and b†), indicating incomplete doping within the sample. However, as shown in Fig. 5d, the strong acid **DCA** (p*K*_a_ = 1.35) caused the doped **TANI(DCA)_2_-PTAB** supra-amphiphile to form well-defined vesicles with an average diameter of 34 nm (Fig. S12†), very similar to those obtained from **TANI(TFA)_2_-PTAB**. In addition, the **TANI(DCA)_2_-PTAB** system exhibits similar doping/de-doping responsive self-assembly behaviour in aqueous solution as found for **TANI(TFA)_2_-PTAB** sample (Fig. S13†).

To ensure that we explored the boundary conditions and limitations of our proposed APP approach, we also considered the use of the very bulky phosphoric acid (*R*)-(–)-1,1′-binaphthyl-2,2′-diyl hydrogen phosphate, **BinPO_4_H**, (*p* = 0.89, p*K*_a_ = 3.37). UV-vis spectra showed that **BinPO_4_H** doped **TANI-PTAB** completely (Fig. S14†). Attempting doping and assembly with this acid produced grain-like structures, which may be considered as aggregated structures of spherical structures (see Fig. 5f and S15†).

Finally, we compared the structures of the oligo(aniline)-based **eSAs** (**HNO_3_**, **DCA**, and **BinPO_4_H**) obtained here with those found for **TANI(CSA)_2_-PTAB** (**CSA**, *p* = 0.73), for which we expected either lamellar or vesicular structures. However, doping with CSA led to the formation of 6 nm-thick wire-like structures.^19^ One aspect of the molecular design and non-covalent interactions we have not considered in any of our discussions are secondary additional interactions between the dopant and the oligo(aniline) moiety. Previous molecular modelling investigations of the successful doping of **PANI** with **CSA**^51^ suggested an exceptionally serendipitous fit of **CSA** into the molecular cavity formed between adjacent phenyl rings (Ph-*N*-Ph), with additional hydrogen-bonding interactions to the **PANI** backbone structure. These postulated fit interactions, in combination with additional hydrogen-bonding capability, could play a significant role in the formation of stable wire-like structures (rather than the expected classical lamellar or vesicular structures). We are currently investigating the perceived anomalous behaviour found for the **CSA**-doped system in more detail.

**Scheme 3:**
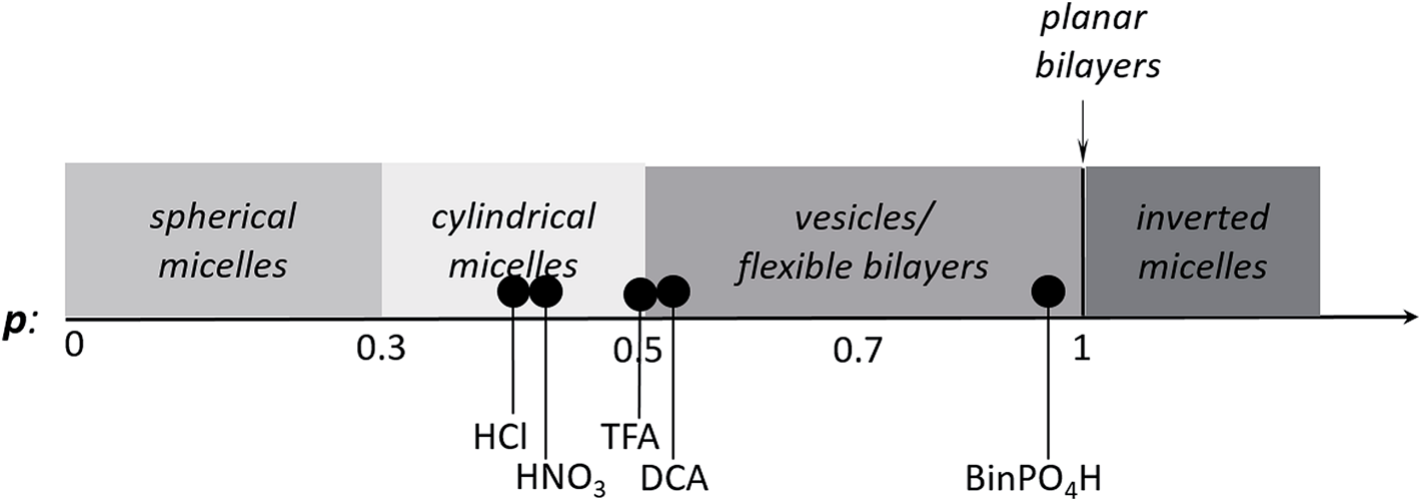
Scheme showing the relationship between packing parameter and the obtained nanostructures using different dopants for our **TANI-PTAB** system.

However, these initial investigations indicate that the strategy employed here, *i.e.*, using a suitable dopant to regulate packing parameters and influence intermolecular assembly forces, is a feasible route produce controllable and tuneable self-assembled structures of oligo(aniline)-based **eSAs** (Scheme 3).

## Conclusion

We have developed a reversibly switchable self-assembling system using an electroactive oligo(aniline)-based **eSA**, **TANI(TFA)_2_-PTAB**, that exhibits dopant-dependent morphology transitions between vesicles and nanowires. This behavior arises from changes in the packing parameter of the protonated oligo(aniline) *vs.* the un-protonated EB form, caused by non-covalent association of the conjugate base as counter-ion to the protonated species. Cycling the system between basic and acidic states allowed reversible switching between nanowires and vesicles, respectively. Further exploration of these addressable self-assembly concepts using acids with different volumes provided packing parameters ranging from 0.41 to 0.89. This reversibly switchable self-assembly behaviour in aqueous solution therefore provides initial design rules that will enable the development of novel switchable and addressable systems for use in encapsulation and delivery, as well as novel biomaterial applications.

## Conflicts of interest

There are no conflicts to declare.

## Supplementary Material

Supplementary information
