# Supplementary material for: An addressable packing parameter approach for reversibly tuning the assembly of oligo(aniline)-based supra-amphiphiles
Source: Chem Sci. 2018 Apr 2;9(19):4392–401. doi: 10.1039/c8sc00068a (PMC5956978; doi:10.1039/c8sc00068a)
Supplement: Supplementary file 1 [file SC-009-C8SC00068A-s001.pdf]

Electronic Supplementary Material (ESI) for Chemical Science.
This journal is © The Royal Society of Chemistry 2018

An addressable packing-parameter approach for reversibly tuning the assembly of oligo(aniline)-based supra-amphiphiles

Wei Lyu,[1,2] Maha Alotaibi,[1,3] O. Alexander Bell,[1] Kazuyoshi Watanabe,[1] Robert Harniman[1], Benjamin. M. Mills,[1] Annela M Seddon,[4,6] Sarah Rogers,[5] Stephen King,[5] Wei Yan,[2] Charl F. J. Faul*,[1]

Supporting information

Table of Contents

SYNTHESIS OF TANI PRESURSOR 2

SYNTHESIS OF TANI-PTAB 4

THEORETICAL ESTIMATION OF PACKING PARAMETER 7

ATTEMPTS TO DETERMINE THE CAC VALUE 18

SCATTERING ANALYSIS 18

DILUTION-DE-DOPING SELF-ASSEMBLY 21

SUPPLEMENTARY ANALYSIS 23

REFERENCES 28

1. Synthesis of TANI precursor

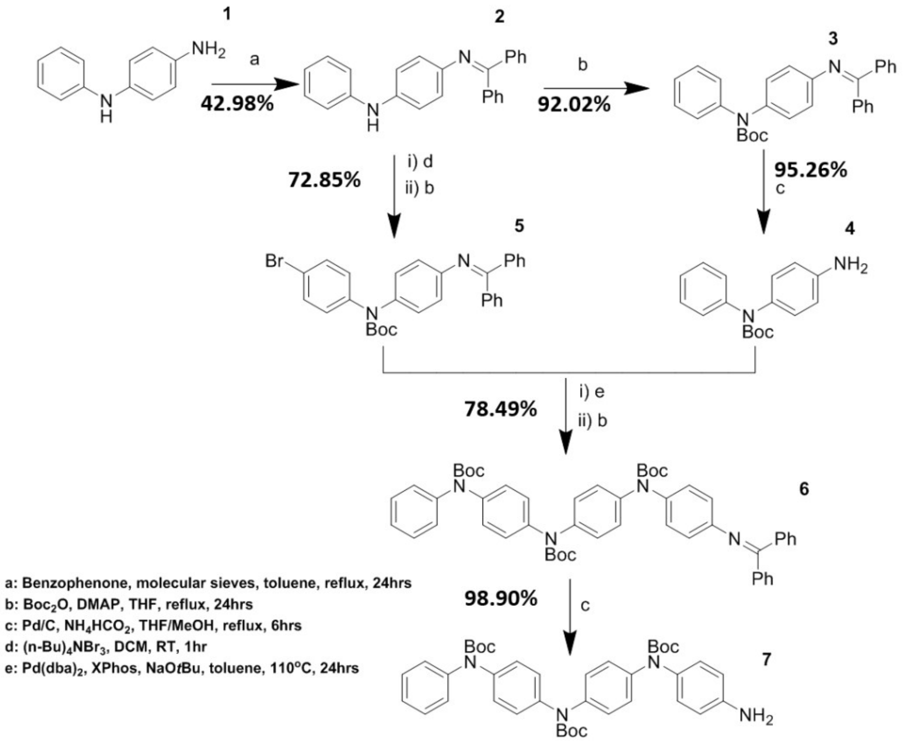

Scheme S1. Synthesis pathway for **TANI** precursor

1.1 Synthesis of 2 (CPH_2 protection)

N-phenyl-p-phenylene diamine (**1**) (1.05 eq, 27.65 g, 150 mmol) and benzophenone (1 eq, 25 g, 135 mmol) were added to a flask containing activated molecular sieves (100 g, 3Å), and the flask placed under N_2. Toluene (150 mL) was added, and the mixture refluxed for 24 hours. Once complete, the mixture was cooled to room temperature and decanted from the molecular sieves. THF was used to wash the sieves until colourless and the combined organic fractions evaporated to give brown oil which solidified into a bright yellow solid when methanol (100 mL) was added. The suspension was filtered and the filtrate concentrated, and filtered again. The combined solids were recrystallized from methanol to yield yellow crystals; with concentration of the mother liquor yielding a second crop (42.98 %).
ESI-TOF-MS: m/z calculated for $C_{25}H_{20}N_2$ 348.2. Found 349.2 $[M+H]^+$, 371.2 $[M+Na]^+$.
^1H NMR (400 MHz, Chloroform-d) δ 7.83-7.75 (m, 2H), 7.58-7.14 (m, 10H), 7.03-6.79 (m, 3H), 6.94-6.86 (m, 2H), 6.76-6.70 (m, 2H), 5.59 (s, 1H).

1.2 Synthesis of 3 (Boc protection)

2 (15.00 g, 42.9 mmol, 1 eq.), di-tert-butyl dicarbonate (11.23 g, 51.3 mmol, 1.2 eq.) and dimethylaminopyridine (0.52 g, 4.29 mmol, 0.1 eq.) were placed under a nitrogen atmosphere and dissolved in anhydrous tetrahydrofuran (200 mL). The solution was refluxed until completion was observed by TLC (4:1 petroleum ether:ethyl acetate) after approximately 24 hrs. On cooling the product precipitated out as fine yellow needles, which were isolated by filtration and then washed with cold ethanol (92.02%).
ESI-TOF-MS: m/z calculated for $C_{30}H_{28}N_2O_2$ 448.2. Found: 449.2 $[M+H]^+$, 471.2 $[M+Na]^+$, 487.2 $[M+K]^+$.
^1H NMR (400 MHz, Chloroform-d) δ 7.75 (d, *J*=7.57, 2H), 7.49-7.39 (m, 3H), 7.35-7.21 (m, 5H), 7.19-7.07 (m, 2H), 6.98 (d, *J*=8.15, 2H), 6.68 (d, *J*=8.20, 2H), 1.41 (s, 9H).

1.3 Synthesis of 4 (CPH_2 deprotection)

3 (10.00 g, 22.3 mmol, 1 eq.), ammonium formate (16.80 g, 0.268 mol, 12 eq.) and palladium on carbon (0.60 g,

0.558 mmol, 2.5 mol%) were added to a flask and placed under nitrogen. Anhydrous tetrahydrofuran (100 mL) and degassed methanol (250 mL) were added and the mixture refluxed for 5 h. Once completion was confirmed by TLC (4:1 petroleum ether:ethyl acetate), the reaction mixture was allowed to cool and the solvent removed under vacuum. The solid was dissolved in dichloromethane (100 mL), filtered through celite and washed through with dichloromethane. The filtrate was evaporated and the residue stirred in n-hexane (300 mL) overnight. Filtration afforded the product as an off-white powder (95.26%).
ESI-TOF-MS: m/z calculated for $C_{17}H_{20}N_2O_2$ 284.2. Found: 307.1 $[M+Na]^+$.
^{1}H NMR (400 MHz, Chloroform-d) δ 7.32-7.09 (m, 5H), 7.04-6.95 (m, 2H), 6.67-6.58 (m, 2H), 3.25 (s, 2H), 1.44 (s, 9H).

1.4 Synthesis of 5 (bromination/Boc protection)

Tetra-n-butylammonium tribromide (18.50 g, 57.4 mmol, 1.1 eq.) was added to a solution of **2** (18.18 g, 52.1 mmol, 1 eq.) in dichloromethane (300 mL) and stirred for 90 minutes at room temperature. An aqueous solution of sodium sulphite (22 wt%, 200 mL) was added and the mixture stirred for a further 30 minutes before addition of sodium hydroxide (2 M, 200 mL). After stirring for a further 10 minutes, the mixture was separated. The organic phase was washed with deionised water, dried over anhydrous $MgSO_4$ then filtered. The solvent was removed before di-tert-butyl dicarbonate (12.51 g, 57.3 mmol, 1.1 eq.) and dimethylaminopyridine (0.64 g, 5.21 mmol, 0.1 eq.) were added. Tetrahydrofuran (250 mL) was added and the mixture refluxed for 24 h. After cooling to room temperature, ethanol (500 mL) was added. The mixture was cooled overnight to yield the product as fine pale-yellow needles, which were isolated by filtration (72.85%).
EA calculated for $C_{30}H_{27}BrN_2O_2$: C 68.31, H 5.16, N 5.31, Br 15.15. Found: C 68.51, H 5.19, N 5.13, Br 14.88.
ESI-TOF-MS: m/z calculated for $C_{30}H_{27}BrN_2O_2$ 527.1. Found 529.1 $[M+2H]^+$, 551.1 $[M+Na]^+$, 567.1 $[M+K]^+$.
^{1}H NMR (400 MHz, Chloroform-d) δ 7.78-7.71 (m, 2H), 7.52-7.21 (m, 8H), 7.16-7.08 (m, 2H), 7.07-6.99 (m, 2H), 6.99-6.90 (m, 2H), 6.72-6.64 (m, 2H), 1.39 (s, 9H).

1.5 Synthesis of 6 (Buchwald-Hartwig/Boc protection)

5 (15.48 g, 29.3 mmol, 1 eq.), **4** (10.00 g, 35.2 mmol, 1.2 eq.), Pd(dba)2 (0.31 g, 0.6 mmol, 2 mol%), XPhos (0.28 g, 0.6 mmol, 2 mol%) and sodium tert-butoxide (5.63 g, 58.6 mmol, 2 eq.) were added to a flask and placed under a nitrogen atmosphere. Anhydrous toluene (300 mL) was added and the mixture refluxed at 110 °C overnight. After completion was observed by TLC (4:1 petroleum ether : ethyl acetate), the mixture was filtered through celite and the filtrate evaporated. The residue was taken up in dichloromethane and washed with water (3x50 mL). The organic phase was dried over anhydrous $MgSO_4$ and the solvent removed under vacuum. Di-tert-butyl dicarbonate (32.00 g, 0.147 mol, 5 eq.) and dimethylaminopyridine (0.36 g, 2.93 mmol, 0.1 eq.) were added to the residue and placed under nitrogen. Anhydrous tetrahydrofuran (300 mL) was added and the mixture refluxed for 3 days, until completion was observed by TLC (4:1 petroleum ether : ethyl acetate). The solvent was removed under vacuum and the residue stirred in methanol then filtered to yield the product as a grey powder (78.49%).
MALDI-TOF-MS: m/z calculated for $C_{52}H_{54}N_4O_6$ 830.4. Found 831.5 $[M+H]^+$.
^{1}H NMR (400 MHz, Chloroform-d) δ 7.78-7.70 (m, 2H), 7.52-7.34 (m, 3H), 7.34-7.07 (m, 18H), 6.95 (d, *J*=8.63, 2H), 6.67 (d, *J*=8.54, 2H), 1.48-1.36 (m, 27H).

1.6 Synthesis of 7 (Benzophenone deprotection)

6 (10.0 g, 12.0 mmol, 1 eq.), ammonium formate (7.59 g, 0.12 mol, 10 eq.) and Pd/C (0.26 g, 0.24 mmol, 2 mol%) were added to a round-bottomed flask and protected under nitrogen. Tetrahydrofuran (100 mL) and methanol (250 mL) were degassed then added to the flask. The mixture was refluxed at 65 °C until completion was observed by TLC (1:1 n-hexane:ethyl acetate) after 6 hours. The solvent was removed and the residue taken up in dichloromethane (400 mL) and filtered through celite. The dichloromethane was removed under vacuum and the solid stirred in hexane overnight to yield the product as a white powder isolated by filtration (98.90%).
ESI-TOF-MS: m/z calculated for $C_{39}H_{46}N_4O_6$ 666.3. Found 689.3 $[M+K]^+$.

^{1}H NMR (400 MHz, DMSO-d6) δ 7.33 (t, *J* = 7.9 Hz, 2H), 7.23-7.05 (m, 11H), 6.83 (d, *J* = 8.5 Hz, 2H), 6.49 (d, *J* = 6.4 Hz, 2H), 5.08 (s, 2H), 1.37-1.31 (m, 27H).

2. Synthesis of TANI-PTAB

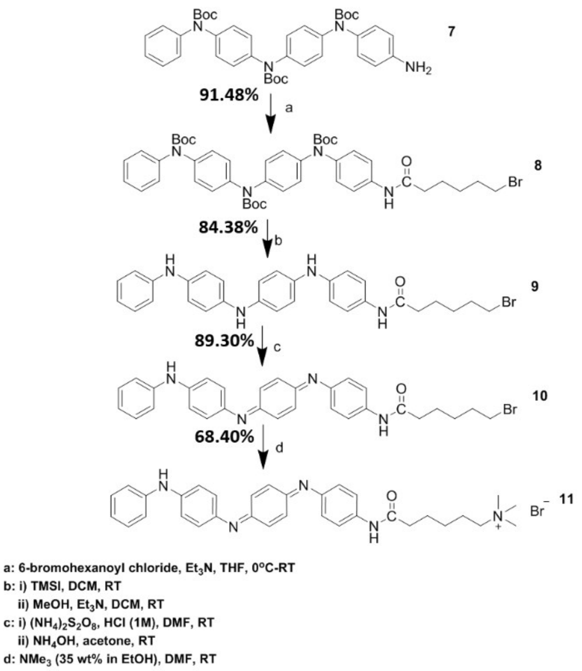

Scheme S2. Synthesis pathway for **TANI-PTAB.**

2.1 Synthesis of 8 (Acylation)

7 (1.0 g, 1.50 mmol, 1 eq.) was added to a flask fitted with a dropping funnel and placed under nitrogen. Anhydrous tetrahydrofuran (35 mL) was added and the solution cooled in ice before trimethylamine (1.04 mL, 7.5 mmol, 5 eq.) was added with stirring. A solution of 6-bromohexanoyl chloride (0.25 mL, 1.50 mmol, 1 eq.) in anhydrous tetrahydrofuran (20 mL) was added added dropwise and the mixture allowed to warm to room temperature. The reaction mixture was stirred overnight and completion was confirmed by TLC (1:1 n-hexane:ethyl acetate). The solvent was removed and the residue taken up in ethyl acetate (50 mL) and filtered to remove precipitated impurities. The solution was then washed with HCl (1M, 35 mL), NaOH (1M, 35 mL) and saturated NaCl (35 mL) then dried over anhydrous $MgSO_4$. The solvent was removed and the residue taken up in ethyl acetate (3 mL) then stirred overnight in n-hexane (35 mL). Filtration yielded the product as an off-white powder (91.48 %).
ESI-TOF-MS: m/z calculated for $C_{45}H_{55}BrN_4O_7$ 842.3. Found 867.3 $[M+Na]^+$.
^{1}H NMR (400 MHz, DMSO-d6) δ 9.94 (s, 1H), 7.53 (d, *J* = 8.6 Hz, 2H), 7.33 (t, *J* = 7.7 Hz, 2H), 7.23 -7.06 (m, 13H), 3.52 (d, *J* = 6.7 Hz, 3H), 2.28 (t, *J* = 7.3 Hz, 2H), 1.80 (p, *J* = 6.8 Hz, 2H), 1.58 (p, *J* = 7.3 Hz, 2H), 1.40 (q, *J* = 8.0 Hz, 2H), 1.34 (s, 27H).

2.2 Synthesis of 9 (Boc deprotection)

A solution of **8** (1.0 g, 1.186 mmol, 1 eq.) in anhydrous dichloromethane (25 mL) was prepared in a flask under a nitrogen atmosphere. Trimethylsilyl iodide (0.61 mL, 4.26 mmol, 3.6 eq.) was added slowly with stirring. After stirring for 2 hours, anhydrous methanol was added dropwise until a maximum pale precipitate was seen (approximately 0.5 mL). Triethylamine (1.6 mL) was added slowly and a colour change to pale purple was observed. The mixture was centrifuged, the supernatant removed and the residue washed 3 times with diethyl ether. The remaining solvent was removed under vacuum to yield the product as an off-white powder (84.38 %).
MALDI-TOF-MS: m/z calculated for $C_{30}H_{31}BrN_4O$ 542.2. Found 542.5 $[M]^+$.
^{1}H NMR (400 MHz, DMSO-d6) δ 9.61 (s, 1H), 7.74 (s, 1H), 7.65 (s, 1H), 7.61 (s, 1H), 7.37 (d, J = 8.6 Hz, 2H), 7.13 (t, *J* =

7.7 Hz, 2H), 7.01-6.83 (m, 13H), 6.66 (t, J = 7.3 Hz, 1H), 3.53 (t, *J* = 6.7 Hz, 2H), 2.24 (t, *J* = 7.3 Hz, 2H), 1.86-1.76 (m, 2H), 1.58 (q, *J* = 7.5 Hz, 2H), 1.41 (q, J = 8.0 Hz, 2H).

2.3 Synthesis of 10 (LEB to EB oxidation)

9 (0.5 g, 0.92 mmol, 1 eq.) was dissolved in a minimum of dimethylformamide (7 mL) and a solution of ammonium persulfate (0.21 g, 0.92 mmol, 1 eq.) in 1M HCl (15 mL) was added dropwise with stirring. A colour change from a pale purple to dark green solution was observed and a precipitate formed. The mixture was stirred for 1 hour then centrifuged and the supernatant removed. The residue was stirred in acetone (100 mL) before ammonium hydroxide (30 mL, 2M, aq.) was added slowly. The mixture turned from green to dark purple and a vapour was produced. After stirring for a further 30 minutes, the acetone was removed under vacuum. The mixture was then centrifuged and the supernatant poured off. The residue was washed 3 times with deionised water (30 mL) then dried under vacuum to yield **10** as a dark-purple powder (89.30 %).

MALDI-TOF-MS: m/z calculated for $C_{30}H_{29}BrN_4O$ 540.2. Found 542.4 $[M+2H]^+$.

^{1}H NMR (400 MHz, DMSO-d6) δ 9.94 (s, 1H), 8.41 (s, 1H), 7.92-6.42 (m, 17H), 3.52 (t, *J* = 6.8 Hz, 2H), 2.29(s, 2H), 1.81 (t, *J* = 7.5 Hz, 2H), 1.58 (d, *J* = 8.3 Hz, 2H), 1.40 (s, 2H).

2.4 Synthesis of 11 (Quaternization)

10 (200 mg, 0.370 mmol, 1 eq.) was dissolved in dimethylformamide (3 mL) and trimethylamine in ethanol (33 wt%, 880 µL, 3.70 mmol, 10 eq.) was added. The solution was stirred at room temperature for 3 days until completion was observed by TLC (ethyl acetate). The mixture was added to diethyl ether (50 mL) and placed in the freezer overnight to precipitate. The mixture was centrifuged and the supernatant removed. The residue was washed 3 times with diethyl ether then dried under vacuum. The resulting purple powder was redissolved in deionised water (20 mL) and freeze-dried to afford the product (68.40 %).

ESI-TOF-MS: m/z calculated for $C_{33}H_{38}BrN_5O$ 599.2. Found 520.3 $[M-Br]^+$ (Figure S1).

^{1}H NMR (400 MHz, Acetonitrile-d3) δ 9.21 (s, 1H), 8.71 (s, 1H), 7.73 (s, 1H), 7.42 (d, *J* =24.7 Hz, 1H), 7.27 (s, 1H), 7.18-6.87 (m, 14H), 3.28 (s, 2H), 3.04 (s, 9H), 2.41 (d, *J* =24.7 Hz, 2H), 1.79 (s, 2H), 1.73 (s, 2H), 1.41 (s, 2H) (Figure S2).

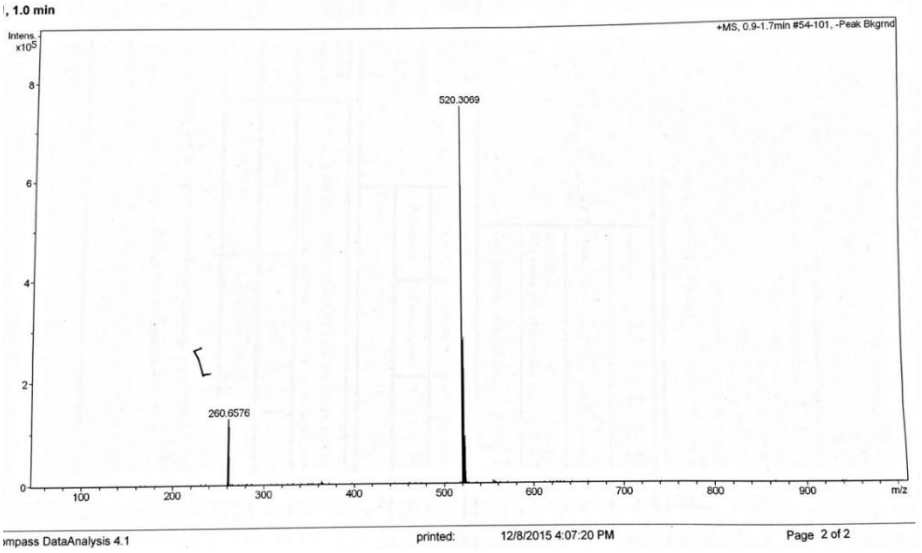

Figure S1. ESI-TOF-MS spectrum of **TANI-PTAB**.

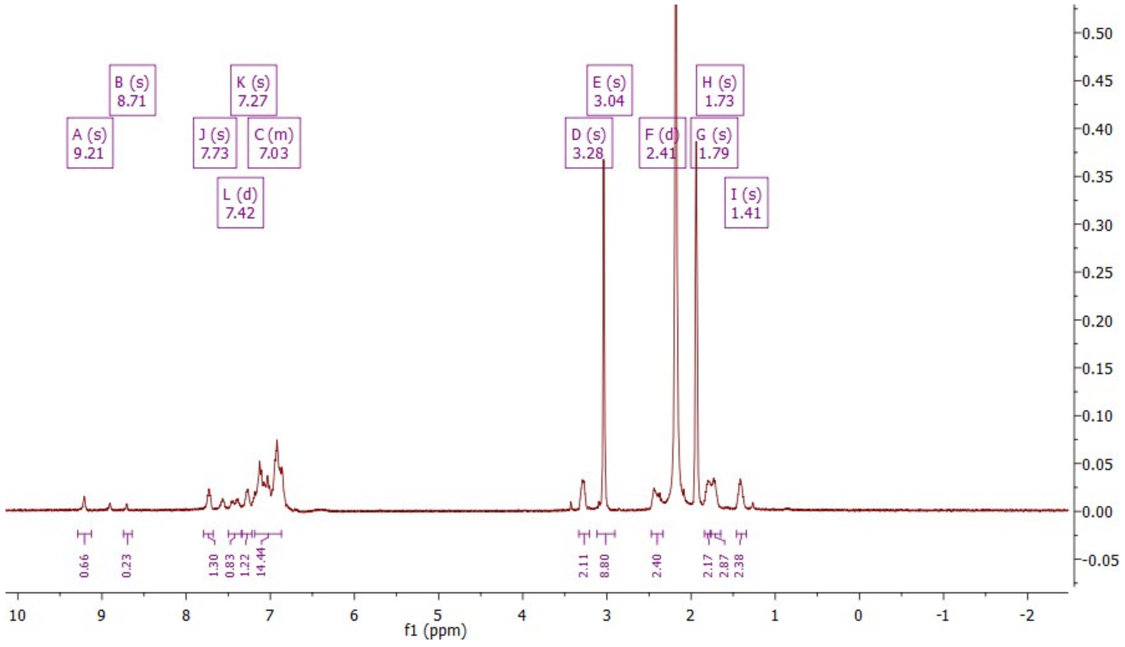

Figure S2. ^{1}H NMR (400 MHz, Acetonitrile-d3) of **TANI-PTAB**.

2. Theoretical estimation of packing parameters for TANI-PTAB and TANI(HX)$_2$-PTAB amphiphilic self-assembled aggregates

Computational details

In this work, **TANI(HX)$_2$-PTAB** structures were achieved by protonation of quinoid N atoms of EB state, a common ES structure for polyaniline.[1] All geometries were optimised using the B3LYP [2-5] functional with a standard 6-31 g (d, p) basis set in Gaussian 09, revision D.01.[6]

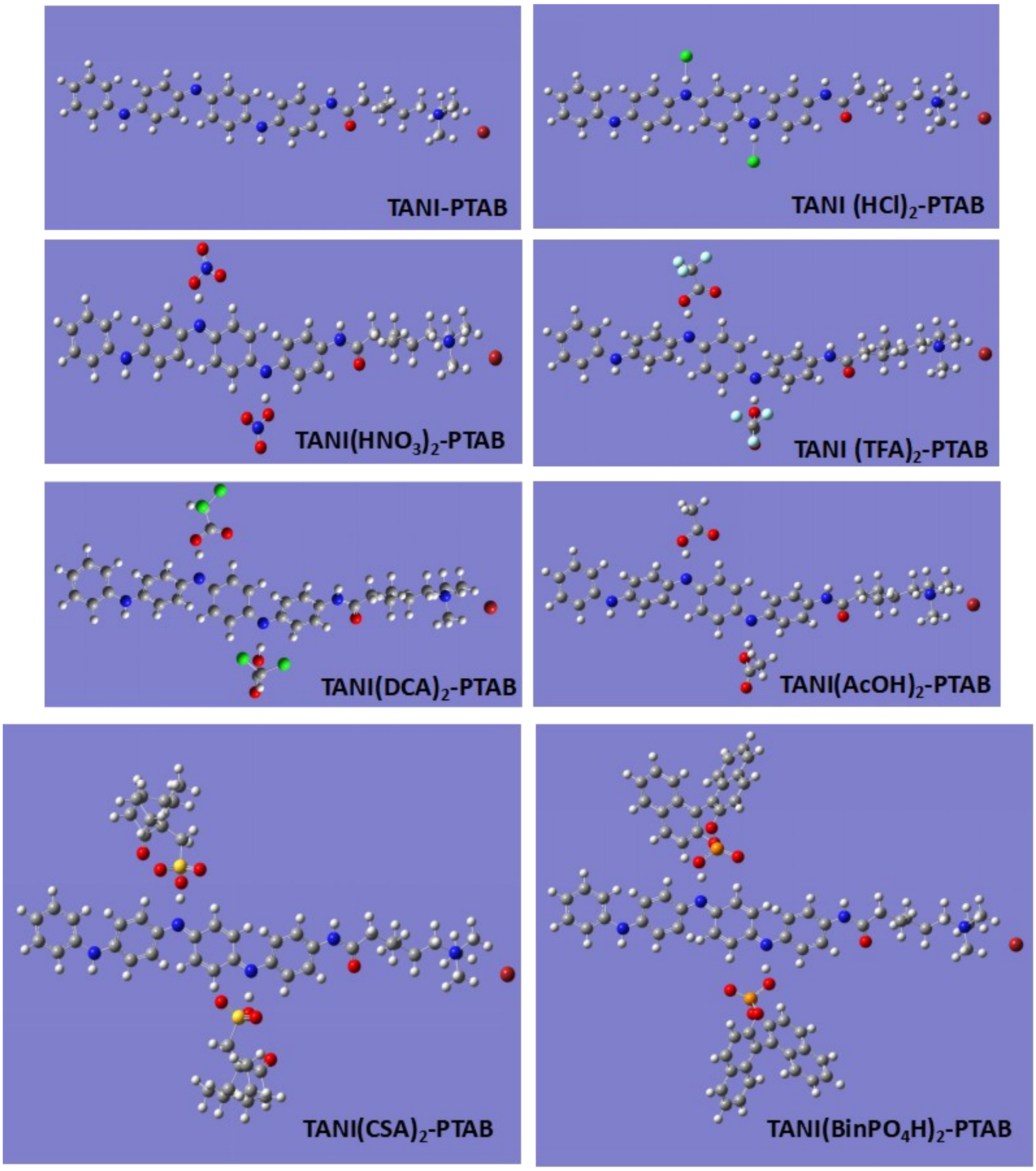

Figure S3. DFT-optimized model of **TANI-PTAB**, **TANI(HCl)$_2$-PTAB, TANI(HNO$_3$)$_2$-PTAB, TANI(TFA)$_2$-PTAB, TANI(DCA)$_2$-PTAB**, **TANI(AcOH)$_2$-PTAB** and **TANI(BinPO$_4$H)$_2$-PTAB**

Table S1. Atomic coordinates for calculated ground state model of EB **TANI-PTAB**, and ES **TANI(HX)$_2$-PTAB** , and corresponding calculated ground-state energy for the structure.

Coordinates of optimized geometry: **TANI-PTAB** E = -4202.031199 a.u.

	X	Y	Z
C	-3.61962868	0.50060859	1.99647805
H	-3.44091429	0.33957231	3.05688537
C	-4.86689194	0.95391971	1.57851734
H	-5.63667054	1.14886486	2.31591064
C	-5.11828586	1.20192630	0.21778669
C	-4.07434936	0.97107534	-0.69537338
H	-4.25108715	1.13499049	-1.75582941
C	-2.83099254	0.51063052	-0.27607363
H	-2.06042000	0.31044416	-1.01239652
C	-2.57911657	0.26877338	1.08425227
N	-6.33559377	1.70051415	-0.25969449
N	-1.35519905	-0.24096474	1.55492647
C	-7.59336914	1.69117710	0.35965237
C	-10.20447156	1.70172585	1.48089152
C	-8.50429216	2.72259363	0.07924450
C	-8.01586330	0.65728643	1.21258549
C	-9.29484015	0.66806538	1.75887291
C	-9.78401155	2.73324129	0.62544204
H	-8.19617415	3.54351798	-0.56420840
H	-7.35416520	-0.17519599	1.42379240
H	-9.60535792	-0.15370288	2.39973088
H	-10.44661158	3.56355633	0.41033802
C	-0.07121048	-0.02517487	1.05898722
C	2.59317023	0.36455531	0.17662157
C	0.25919559	1.05913193	0.22607605
C	0.96460220	-0.89876871	1.43802490
C	2.27708052	-0.71204639	1.01884854
C	1.56756412	1.23784652	-0.20907654
H	-0.50154667	1.77314892	-0.06640476
H	0.73400094	-1.74731911	2.07807193
H	3.05598820	-1.39536140	1.32590866
H	1.79469688	2.08664307	-0.85156963
N	3.89815416	0.62584089	-0.31016802
N	-11.46333878	1.67805220	2.10310259
H	3.97410027	1.44766522	-0.89329917
H	-11.48181458	1.25203979	3.01741586
C	-12.64834857	2.30802602	1.71631480
C	-15.11388263	3.49430113	1.02347944
C	-13.60685203	2.61039002	2.70170014
C	-12.95051894	2.59993942	0.37334432
C	-14.16727428	3.19370818	0.04215396
C	-14.82491815	3.18831796	2.35531681
H	-13.38461801	2.39224366	3.74391449
H	-12.24463924	2.33978850	-0.40705010
H	-14.38044619	3.40906980	-1.00147541
H	-15.54803939	3.41004507	3.13541409
H	-16.05985698	3.95416033	0.75559669
C	5.06539822	-0.04881465	-0.06600502
O	5.14892324	-1.05735627	0.62778784
C	6.29730245	0.59231350	-0.72143013
H	6.49506271	1.54171035	-0.20568497
H	6.07061710	0.85244171	-1.76442328
C	7.53762481	-0.30472087	-0.64219733
H	8.41417491	0.29955589	-0.90548915
H	7.66044463	-0.62401535	0.39857014
C	7.44556591	-1.54827127	-1.54079827
H	7.40556392	-1.24410053	-2.59659911
H	6.50436840	-2.06096723	-1.32255675
C	8.58268442	-2.56897684	-1.33977584
H	8.34996491	-3.46564515	-1.92302621
H	8.60353141	-2.86675911	-0.28599351
C	9.93950579	-2.00667047	-1.76379161
N	11.12359164	-2.95146665	-1.66489269
H	9.90452357	-1.68323859	-2.80926045
H	10.20694440	-1.13932762	-1.15457160
C	12.36646299	-2.21246338	-2.11226777
H	13.20927433	-2.91780486	-2.03160681
H	12.50386540	-1.34894315	-1.45910074
H	12.21687390	-1.88484487	-3.14265356
C	11.34832834	-3.42338717	-0.24652558
H	12.26261296	-4.03796852	-0.25908807
H	10.48855227	-4.01220654	0.07069351
H	11.46219358	-2.54519364	0.39212884
C	10.95717836	-4.16007606	-2.55795093
H	10.78011268	-3.81067528	-3.57711371
H	10.10964993	-4.74770292	-2.20699437
Br	14.13392416	-5.15059466	-1.48340308
H	11.89103782	-4.74037372	-2.48296419
H	-6.28020061	2.22674969	-1.11824371
H	-1.41420466	-0.84729460	2.35868319

Coordinates of optimized geometry: **TANI(HCl)$_2$-PTAB** E=-5122.41920222

	X	Y	Z
C	3.11378700	2.92448600	-0.18192800
H	2.88757000	3.97689000	-0.33394700
C	4.39029600	2.45735200	-0.19596600
H	5.21102500	3.15296300	-0.31549800
C	4.66327300	1.06123400	0.03007900
C	3.55078800	0.17816000	0.30000200
H	3.77590300	-0.87339100	0.45832000
C	2.27265500	0.64266000	0.30875600
H	1.45149600	-0.05072800	0.43655200
C	1.99851500	2.03922300	0.08212900
N	5.87565900	0.50475600	0.03475900
N	0.78898800	2.59231700	0.08071300
C	7.09562000	0.99538100	-0.38501200
C	9.68842800	1.80837500	-1.16862500
C	8.24929500	0.38755500	0.17229400
C	7.26627400	1.99565000	-1.37307800
C	8.53194200	2.38829200	-1.75168400
C	9.51448300	0.79511100	-0.19507200
H	8.11121600	-0.39576500	0.91207500
H	6.40948800	2.40926000	-1.89076900
H	8.64944800	3.12898500	-2.53808900
H	10.37689100	0.34939400	0.28372200
C	-0.43832800	2.06347800	0.47689800
C	-3.00415900	1.16766700	1.19917300
C	-0.59235000	1.10239100	1.49644700
C	-1.58964800	2.60414900	-0.13349700
C	-2.85671500	2.15756700	0.20934300
C	-1.85855700	0.66373500	1.84477000
H	0.26535100	0.74269800	2.05189800
H	-1.46243500	3.38084900	-0.88179700
H	-3.73353500	2.56494600	-0.27228900
H	-1.96608100	-0.06595900	2.64364300
N	-4.24577400	0.65380000	1.60293100
N	10.91863200	2.26913100	-1.56981200
H	-4.20142700	-0.05028400	2.32720800
H	10.90867700	3.13691600	-2.08779600
C	12.20240700	1.73026000	-1.33094500
C	14.80264900	0.75765300	-0.93866700
C	13.27552000	2.62022400	-1.17030500
C	12.44220500	0.34765400	-1.31513900
C	13.73636200	-0.12749100	-1.10736300
C	14.56648000	2.13357400	-0.98125000
H	13.08999800	3.69114000	-1.18233800
H	11.63019100	-0.34836600	-1.49216600
H	13.91103000	-1.19911100	-1.09614800
H	15.38769000	2.83298900	-0.85706000
H	15.80803400	0.37963600	-0.78310500
C	-5.51419800	0.98827900	1.16404000
O	-5.73554200	1.82137800	0.29853900
C	-6.62996500	0.22537400	1.88401800
H	-6.64396300	0.56462800	2.92858900
H	-6.38225600	-0.84460400	1.91376300
C	-8.00609000	0.44715400	1.24638300
H	-8.76482700	0.07469800	1.94485500
H	-8.16614000	1.52542100	1.13803100
C	-8.15482900	-0.22560500	-0.12747800
H	-8.04784700	-1.31477100	-0.02233400
H	-7.33529200	0.11191500	-0.76809200
C	-9.47397700	0.09495500	-0.85771200
H	-9.40470300	-0.29238700	-1.87886900
H	-9.57985300	1.18237100	-0.93324500
C	-10.68312500	-0.51632600	-0.14962100
N	-12.03198700	-0.30570200	-0.81231600
H	-10.56020700	-1.60086100	-0.06446900
H	-10.78857600	-0.11218300	0.86053700
C	-13.09052300	-0.98165500	0.03195900
H	-14.05902300	-0.81491800	-0.46428700
H	-13.07135900	-0.53500200	1.02764500
H	-12.85287400	-2.04519800	0.09223500
C	-12.38344600	1.16100100	-0.91405700
H	-13.40002500	1.21604000	-1.33347800
H	-11.66714400	1.65271500	-1.57116000
H	-12.33843400	1.59456600	0.08693400
C	-12.08195900	-0.91502300	-2.19471400
H	-11.81506400	-1.97051200	-2.11342200
H	-11.37478100	-0.39405000	-2.83893300
Br	-15.42825600	0.12628400	-2.32475200
H	-13.11186600	-0.78445000	-2.56196000
H	5.92428500	-0.53536400	0.48135300
H	0.73371600	3.63471300	-0.34925100
Cl	0.68885600	5.22165400	-1.10736200
Cl	5.99241700	-2.12103200	1.22830000

Coordinates of optimized geometry: **TANI(HNO_3)$_2$-PTAB** E = -4762.58811575 a.u.

	X	Y	Z
C	3.36133000	2.83155300	0.00407800
H	3.27069900	3.91047600	0.07628300
C	4.57515100	2.23895400	-0.09347100
H	5.47141500	2.84753000	-0.10132600
C	4.70897800	0.78920400	-0.11253200
C	3.48661600	0.01721000	0.04933500
H	3.58137200	-1.06245500	0.05476300
C	2.27109400	0.60746400	0.14482800
H	1.37780200	-0.00323100	0.19253300
C	2.13364200	2.05696500	0.12876200
N	5.83578900	0.11759200	-0.23872200
N	1.00473100	2.72768300	0.20559500
C	7.04981000	0.68123600	-0.62138100
C	9.61623300	1.63308600	-1.38863600
C	8.23266800	0.11795900	-0.09216900
C	7.18857600	1.71065000	-1.58218100
C	8.43861100	2.16578700	-1.96010900
C	9.48394200	0.59696800	-0.43813400
H	8.14870500	-0.68692600	0.62995300
H	6.30991900	2.10399400	-2.08129000
H	8.52043700	2.92841900	-2.73042700
H	10.36393000	0.18027200	0.03549500
C	-0.22822900	2.16023900	0.54668800
C	-2.79791700	1.19917600	1.22258300
C	-0.39393800	1.20859100	1.57409900
C	-1.38357700	2.65112700	-0.09285300
C	-2.64890900	2.17012300	0.21661200
C	-1.65779300	0.74151100	1.90427800
H	0.46464400	0.87292800	2.14523800
H	-1.27612100	3.41268100	-0.85828400
H	-3.52315500	2.54218300	-0.29754700
H	-1.76382100	0.02090100	2.71214900
N	-4.04153500	0.66137300	1.60541900
N	10.83380700	2.16289300	-1.77434100
H	-3.99758300	-0.03034300	2.34124100
H	10.78703400	3.06128200	-2.23294600
C	12.13431200	1.65827900	-1.58089900
C	14.77876800	0.75612100	-1.28067400
C	13.18860300	2.57449400	-1.43442800
C	12.41933800	0.28331900	-1.59352800
C	13.73285000	-0.15582200	-1.43193000
C	14.49867800	2.12431600	-1.29322900
H	12.97234600	3.63985300	-1.42275400
H	11.62402100	-0.43481800	-1.75713000
H	13.93838800	-1.22226500	-1.44354600
H	15.30155800	2.84718700	-1.18194600
H	15.79910700	0.40595400	-1.16111700
C	-5.30239000	0.96366600	1.13619500
O	-5.52496200	1.78425000	0.25662600
C	-6.41968700	0.18288100	1.83708200
H	-6.45251200	0.51305500	2.88406900
H	-6.15989100	-0.88439900	1.86167400
C	-7.78958900	0.39297100	1.18256700
H	-8.55430500	0.01061800	1.86920900
H	-7.95854500	1.47006000	1.07556700
C	-7.91395500	-0.27551800	-0.19591600
H	-7.80668500	-1.36487600	-0.09272300
H	-7.08402400	0.06637800	-0.82046100
C	-9.22088600	0.04566400	-0.94717000
H	-9.13372100	-0.33593500	-1.96919000
H	-9.32750400	1.13330700	-1.01859700
C	-10.44116700	-0.57146200	-0.26379100
N	-11.77927000	-0.35418000	-0.94545400
H	-10.31972400	-1.65680900	-0.18674400
H	-10.56206100	-0.17678300	0.74841400
C	-12.85113700	-1.03998400	-0.12605600
H	-13.81173400	-0.86783200	-0.63717900
H	-12.84804600	-0.60415400	0.87463800
H	-12.61312900	-2.10391900	-0.07363300
C	-12.13044900	1.11335000	-1.03637600
H	-13.14040100	1.17145100	-1.47278000
H	-11.40338300	1.61290300	-1.67556700
H	-12.10250700	1.53514900	-0.02972400
C	-11.80714900	-0.94776800	-2.33523200
H	-11.54141600	-2.00414500	-2.26130800
H	-11.08929400	-0.41974600	-2.96167900
Br	-15.14257100	0.09323400	-2.50296200
H	-12.83172200	-0.81180700	-2.71711900
O	6.36809800	-2.49753800	-0.12332000
H	5.99734900	-1.53971100	-0.07333500
H	0.90781400	4.29686200	-0.24598300
O	0.64791400	5.19459600	-0.68773400
N	1.34484300	6.19069700	-0.06567000
O	2.18150100	5.85474400	0.76864100
O	1.06231900	7.31489000	-0.41579300
N	5.59535800	-3.29615000	0.67363800
O	5.96144300	-4.44709300	0.75463100
O	4.62381000	-2.77894900	1.21710100

Coordinates of optimized geometry: **TANI(TFA)$_2$-PTAB** E = -5254.390085 a.u.

	X	Y	Z
C	3.31270300	2.85109800	-0.01553500
H	3.21420700	3.93001700	0.01536200
C	4.53162400	2.26994200	-0.11234600
H	5.42108500	2.88705700	-0.15308800
C	4.67949500	0.82150800	-0.08373600
C	3.46591600	0.03959300	0.09940000
H	3.56395700	-1.04257900	0.10339300
C	2.24557500	0.62264700	0.18597100
H	1.35804400	0.00430800	0.24350300
C	2.09342000	2.06973000	0.14549300
N	5.81623300	0.16099500	-0.18249600
N	0.96346100	2.73649900	0.22512800
C	7.02132900	0.72350300	-0.59349400
C	9.56839200	1.67757600	-1.42018600
C	8.21528800	0.20627000	-0.04218100
C	7.13914100	1.70716100	-1.60367900
C	8.37992600	2.16339800	-2.01023800
C	9.45685800	0.68784400	-0.41892000
H	8.14897400	-0.56116200	0.72148000
H	6.25171600	2.06125000	-2.11669700
H	8.44566600	2.88848800	-2.81745700
H	10.34587600	0.30942200	0.06988900
C	-0.27092300	2.17805300	0.56833400
C	-2.85271300	1.24138400	1.22681800
C	-0.44924400	1.21590500	1.58319200
C	-1.41865800	2.69144600	-0.06715200
C	-2.69033000	2.22251100	0.23254800
C	-1.71975400	0.76150000	1.90537900
H	0.40341600	0.86352800	2.15284800
H	-1.29505800	3.45988600	-0.82350000
H	-3.55902600	2.61089400	-0.27896000
H	-1.83699500	0.03268600	2.70427700
N	-4.10335800	0.71477100	1.60145500
N	10.77619600	2.20628500	-1.83765200
H	-4.06927300	0.01469200	2.32984500
H	10.71397100	3.08027000	-2.33951400
C	12.08437700	1.72658000	-1.63352300
C	14.74253300	0.87025100	-1.31846100
C	13.12897500	2.66079400	-1.54083600
C	12.38598000	0.35597200	-1.58416500
C	13.70622800	-0.05982600	-1.41583300
C	14.44572900	2.23297800	-1.39222500
H	12.89998100	3.72293100	-1.57701300
H	11.59789000	-0.37834000	-1.70540100
H	13.92450800	-1.12318900	-1.37948600
H	15.24083700	2.96958600	-1.32328500
H	15.76816400	0.53799200	-1.19323500
C	-5.35974000	1.03724700	1.13338600
O	-5.57092900	1.86838500	0.26104500
C	-6.48760800	0.26503800	1.82666500
H	-6.52397100	0.59542900	2.87348700
H	-6.23708200	-0.80439900	1.85257600
C	-7.85178700	0.48697500	1.16422800
H	-8.62342500	0.10840900	1.84518800
H	-8.01219300	1.56553600	1.05906700
C	-7.97234500	-0.17695200	-0.21674100
H	-7.86923700	-1.26697600	-0.11636600
H	-7.13839200	0.16385400	-0.83656600
C	-9.27496600	0.15054300	-0.97277200
H	-9.18436500	-0.22826100	-1.99554400
H	-9.37829500	1.23869300	-1.04141000
C	-10.49985800	-0.46521000	-0.29640600
N	-11.83409200	-0.24558500	-0.98486100
H	-10.38048200	-1.55080500	-0.21952800
H	-10.62548400	-0.07112300	0.71545600
C	-12.91130800	-0.92869200	-0.17022400
H	-13.86902300	-0.75553600	-0.68644900
H	-12.91260900	-0.49173700	0.82998200
H	-12.67527400	-1.99294900	-0.11538700
C	-12.18181600	1.22257700	-1.07862900
H	-13.18957000	1.28239000	-1.51980300
H	-11.45068600	1.72009000	-1.71478000
H	-12.15773500	1.64517600	-0.07221100
C	-11.85631100	-0.84019200	-2.37428400
H	-11.59314600	-1.89706000	-2.29822700
H	-11.13423400	-0.31418400	-2.99754600
Br	-15.18974100	0.20597900	-2.55774800
H	-12.87869100	-0.70247300	-2.76135600
O	6.35567100	-2.38183200	0.43716600
H	6.00031000	-1.45122300	0.18993900
C	5.40904000	-3.28997800	0.51814400
O	4.21730500	-3.14518200	0.34313400
C	6.00964500	-4.66516400	0.89143700
F	6.91375000	-5.05129700	-0.02781600
F	5.05414700	-5.59632500	0.96421800
F	6.62985200	-4.59581800	2.08508900
H	0.90350900	4.32122800	-0.21617500
O	0.83576700	5.23765700	-0.66585100
C	0.80369400	6.28563500	0.14211000
O	0.67879000	7.42942500	-0.21731900
C	0.97082600	5.94721400	1.64595600
F	0.02998500	5.06510100	2.04911100
F	2.18189100	5.37335800	1.86157900
F	0.88387700	7.03510800	2.40761600

Coordinates of optimized geometry: **TANI(DCA)$_2$-PTAB** E = -6497.316602 a.u.

	X	Y	Z
C	3.31533300	2.89560500	-0.02210700
H	3.23663800	3.97675300	-0.02114300
C	4.51932300	2.29093300	-0.14944500
H	5.41754100	2.89124600	-0.22771100
C	4.64109900	0.83902000	-0.11374000
C	3.41478200	0.08054900	0.09070300
H	3.49353800	-1.00312800	0.09759500
C	2.20914500	0.68711600	0.20304200
H	1.31143900	0.08633400	0.28254700
C	2.08483100	2.13903800	0.18021500
N	5.76162400	0.15685600	-0.22751400
N	0.97267300	2.82483300	0.31038700
C	6.97582500	0.70071300	-0.64367700
C	9.53504500	1.61453900	-1.47698600
C	8.16136900	0.18068200	-0.07977300
C	7.10669000	1.66548400	-1.66892900
C	8.35420100	2.10180900	-2.07935100
C	9.40989400	0.64351200	-0.45995000
H	8.08306700	-0.57237200	0.69717000
H	6.22400900	2.02231800	-2.18844500
H	8.42987500	2.81380200	-2.89732100
H	10.29316700	0.26531200	0.03949500
C	-0.26282300	2.25638000	0.64548800
C	-2.83741300	1.28738300	1.27808900
C	-0.43913800	1.30184600	1.66633900
C	-1.40769500	2.74652600	-0.01045100
C	-2.67663500	2.26054200	0.27596600
C	-1.70740200	0.83151900	1.97677900
H	0.41294900	0.96501800	2.24645600
H	-1.28290600	3.51013600	-0.77152600
H	-3.54401600	2.62984000	-0.25171900
H	-1.82409100	0.10732200	2.77995600
N	-4.08534400	0.74353400	1.64035400
N	10.75075900	2.12629000	-1.89799000
H	-4.04916500	0.04934400	2.37422500
H	10.69883100	2.99795500	-2.40481700
C	12.05266600	1.63657600	-1.68448300
C	14.70234700	0.75730800	-1.35095200
C	13.10812000	2.56051400	-1.60958100
C	12.34011400	0.26399400	-1.60780300
C	13.65569800	-0.16276700	-1.43064600
C	14.42004400	2.12137300	-1.45193100
H	12.89104100	3.62425900	-1.66677600
H	11.54415600	-0.46394100	-1.71490500
H	13.86227900	-1.22759700	-1.37318100
H	15.22297900	2.85069000	-1.39711100
H	15.72422500	0.41644500	-1.21839000
C	-5.33960500	1.03991500	1.15242400
O	-5.55418400	1.86147000	0.27143000
C	-6.46301400	0.25101100	1.83477200
H	-6.51494400	0.58022300	2.88129000
H	-6.19661100	-0.81454300	1.86283500
C	-7.82371200	0.45197700	1.15884100
H	-8.59640000	0.06320700	1.83287100
H	-7.99885800	1.52793400	1.05018500
C	-7.92129300	-0.21587300	-0.22212700
H	-7.80288500	-1.30407900	-0.11890100
H	-7.08655800	0.13654200	-0.83431100
C	-9.22140400	0.09083100	-0.99092100
H	-9.11575600	-0.28844700	-2.01209600
H	-9.34004100	1.17720400	-1.06252200
C	-10.44343700	-0.54154200	-0.32481000
N	-11.77360300	-0.34603600	-1.02839800
H	-10.30783400	-1.62481200	-0.24215700
H	-10.58598900	-0.14534800	0.68398900
C	-12.84900900	-1.04068200	-0.22122400
H	-13.80358000	-0.88471400	-0.74849500
H	-12.86741200	-0.59831100	0.77642000
H	-12.59776300	-2.10097800	-0.15779900
C	-12.14252100	1.11612100	-1.13420100
H	-13.14646300	1.15805800	-1.58600600
H	-11.41247600	1.62115900	-1.76566500
H	-12.13488300	1.54452000	-0.12997200
C	-11.77198100	-0.94894300	-2.41428800
H	-11.49461500	-2.00149000	-2.32902800
H	-11.05038700	-0.41647600	-3.03256300
Br	-15.11927600	0.04749100	-2.63999300
H	-12.79177400	-0.82829200	-2.81366700
O	6.27635200	-2.42293000	0.32093100
H	5.92898800	-1.48424400	0.11127600
C	5.30041600	-3.29599900	0.49796600
O	4.11065200	-3.09418900	0.38616700
H	0.98337100	4.49617700	0.01553200
O	1.04536100	5.39609200	-0.44969700
C	1.00778700	6.49683100	0.28269100
O	1.08198000	7.60988200	-0.18514100
C	0.86594300	6.37101200	1.81488700
H	0.86214500	7.37345600	2.23191600
C	5.90080300	-4.65882200	0.89845800
H	6.88285800	-4.78811900	0.44991300
Cl	-0.69047500	5.59288300	2.27430600
Cl	2.27783900	5.50622200	2.53934700
Cl	6.15481600	-4.66155900	2.68788900
Cl	4.87101500	-6.01455400	0.37456800

Coordinates of optimized geometry: **TANI(AcOH)$_2$-PTAB** E = -4658.989002 a.u.

	X	Y	Z
C	3.33155400	2.86298100	-0.08146200
H	3.21518500	3.94152700	-0.11626700
C	4.55383600	2.29051600	-0.17859300
H	5.43557700	2.91444300	-0.26152500
C	4.71546100	0.84288100	-0.10819800
C	3.50926900	0.05499000	0.11141100
H	3.62513000	-1.02561800	0.15590900
C	2.28510000	0.62938200	0.19429300
H	1.40418200	0.00526500	0.28454200
C	2.11993400	2.07557100	0.12387300
N	5.84925600	0.18300900	-0.20334600
N	0.99282600	2.74180800	0.21690300
C	7.05324200	0.73735000	-0.62552100
C	9.60678600	1.66973600	-1.45910000
C	8.24388200	0.22380800	-0.06467800
C	7.17824400	1.70305600	-1.65167000
C	8.42291500	2.14784200	-2.06209000
C	9.48905100	0.69646700	-0.44361700
H	8.16863400	-0.53591800	0.70603100
H	6.29401800	2.05282800	-2.17311200
H	8.49400100	2.85996700	-2.88044500
H	10.37541300	0.32257000	0.05370800
C	-0.23884500	2.17942300	0.56385100
C	-2.82620700	1.25336500	1.22775200
C	-0.42311600	1.23411200	1.59341000
C	-1.38540300	2.67968600	-0.08473900
C	-2.65964700	2.21689200	0.21761500
C	-1.69613300	0.78524000	1.91784000
H	0.42780300	0.88549800	2.16819000
H	-1.25489700	3.43314900	-0.85554700
H	-3.52613300	2.59648000	-0.30434600
H	-1.81629800	0.06723600	2.72627500
N	-4.07962400	0.73079300	1.60497700
N	10.81930300	2.19284900	-1.87901400
H	-4.04668600	0.03326600	2.33576600
H	10.76021100	3.06805900	-2.37880900
C	12.12382800	1.70702100	-1.67940200
C	14.78079800	0.83643500	-1.37428000
C	13.17765700	2.63414800	-1.61627700
C	12.41712800	0.33532300	-1.60468500
C	13.73605400	-0.08678000	-1.44199700
C	14.49259500	2.19948600	-1.47274900
H	12.95662400	3.69720000	-1.67183600
H	11.62261500	-0.39544800	-1.70225600
H	13.94654700	-1.15099800	-1.38636400
H	15.29356100	2.93166600	-1.42713900
H	15.80521300	0.49897800	-1.25286500
C	-5.33395100	1.05379500	1.13582400
O	-5.54463900	1.88403500	0.26178600
C	-6.46401900	0.28404000	1.82925000
H	-6.50206800	0.61615200	2.87547000
H	-6.21502600	-0.78569100	1.85733200
C	-7.82688300	0.50638700	1.16431600
H	-8.60014900	0.12877600	1.84404300
H	-7.98625700	1.58499100	1.05800300
C	-7.94528800	-0.15803300	-0.21658500
H	-7.84133000	-1.24796600	-0.11589700
H	-7.11081000	0.18356000	-0.83520500
C	-9.24736300	0.16804900	-0.97418900
H	-9.15504500	-0.21004900	-1.99708000
H	-9.35218400	1.25609200	-1.04230800
C	-10.47201300	-0.44996800	-0.29952500
N	-11.80616900	-0.23149500	-0.98859200
H	-10.35123400	-1.53548800	-0.22377900
H	-10.59888600	-0.05720700	0.71268900
C	-12.88272800	-0.91767800	-0.17574900
H	-13.84046200	-0.74527900	-0.69216000
H	-12.88525500	-0.48242300	0.82519700
H	-12.64497900	-1.98163900	-0.12254900
C	-12.15629600	1.23620600	-1.08030700
H	-13.16378400	1.29508400	-1.52214000
H	-11.42544200	1.73595600	-1.71501400
H	-12.13371200	1.65721600	-0.07319300
C	-11.82638100	-0.82389400	-2.37895000
H	-11.56134500	-1.88040400	-2.30440200
H	-11.10474000	-0.29563600	-3.00080700
Br	-15.16242400	0.21624100	-2.56259900
H	-12.84869300	-0.68744700	-2.76656300
O	6.42329400	-2.45733500	0.44698000
H	6.05162500	-1.55800500	0.20584900
C	5.44482700	-3.34909400	0.62203900
O	4.25775300	-3.09457100	0.50568400
C	5.98235500	-4.71637200	0.97934300
H	1.01124100	4.48518700	-0.19864000
O	1.14024600	5.41130200	-0.54089200
C	0.82407100	6.35656800	0.36823400
O	0.95125100	7.53195100	0.11215900
C	0.30523000	5.84183400	1.70275400
H	1.03561000	5.17320900	2.17026600
H	0.10776600	6.68739100	2.36096400
H	-0.61522700	5.26596900	1.55956900
H	6.63074500	-5.08128900	0.17695100
H	6.59253700	-4.65294300	1.88528000
H	5.15571000	-5.40918400	1.13300800

Coordinates of optimized geometry: **TANI(CSA)$_2$-PTAB** E = -6378.84191460 a.u.

	X	Y	Z
C	2.27556800	3.29527000	0.90453900
H	2.12600900	4.36381300	0.79076100
C	3.52215700	2.78025400	1.02616900
H	4.38071300	3.44026400	1.01772100
C	3.72834400	1.35710800	1.24979900
C	2.53427400	0.53532600	1.38125700
H	2.67942600	-0.52699500	1.54062100
C	1.28786000	1.04899700	1.25587700
H	0.42636900	0.39381700	1.29233800
C	1.08117700	2.47023900	1.02326000
N	4.88987000	0.74658200	1.37414800
N	-0.07849000	3.07624500	0.90077400
C	6.14001100	1.28229400	1.09817300
C	8.78209400	2.18470200	0.58676600
C	7.23396400	0.81253600	1.86630400
C	6.40912100	2.18625100	0.04387300
C	7.69880800	2.61692900	-0.21002700
C	8.51754100	1.27746000	1.63565800
H	7.06906500	0.12216000	2.69147800
H	5.61078500	2.49207800	-0.62281700
H	7.88990600	3.27612600	-1.05337900
H	9.32138400	0.94995400	2.28271200
C	-1.31403200	2.49522500	1.19790200
C	-3.89145300	1.48472900	1.73807700
C	-1.53803100	1.66032900	2.31193100
C	-2.41882000	2.85296500	0.40087400
C	-3.68650500	2.34169800	0.64329400
C	-2.80711300	1.16770500	2.57375500
H	-0.72852100	1.44386700	2.99991700
H	-2.26742200	3.53648700	-0.42723800
H	-4.52020500	2.61026800	0.01110500
H	-2.96390000	0.54048100	3.44856700
N	-5.14314400	0.93066300	2.07070600
N	10.04269400	2.68523000	0.30592700
H	-5.14335800	0.35682200	2.90286100
H	10.05555500	3.52660100	-0.25196600
C	11.30197400	2.25628100	0.76475900
C	13.87772800	1.48977500	1.58916500
C	12.31042100	3.21764400	0.94315300
C	11.60211500	0.90166000	0.98566100
C	12.87871400	0.53185100	1.40625200
C	13.58743200	2.83384100	1.34464400
H	12.08284500	4.26756400	0.77513200
H	10.85011700	0.14257300	0.80394100
H	13.09536300	-0.51929900	1.57415900
H	14.35432900	3.59153400	1.47751400
H	14.87057400	1.19248300	1.91163200
C	-6.36162300	1.08711700	1.44761200
O	-6.53560300	1.76203600	0.44195900
C	-7.50285000	0.34106700	2.14997600
H	-7.58722200	0.73312900	3.17227800
H	-7.22870600	-0.71821300	2.25210000
C	-8.84590400	0.48266600	1.42500000
H	-9.63474800	0.14921700	2.11020200
H	-9.02069300	1.54550200	1.22364400
C	-8.91150600	-0.29704600	0.10194600
H	-8.79426500	-1.37273600	0.29766100
H	-8.06322600	0.00748500	-0.51634000
C	-10.19566500	-0.05983900	-0.71700400
H	-10.06751000	-0.52064900	-1.70143400
H	-10.31597700	1.01655300	-0.87982900
C	-11.42876100	-0.64261700	-0.02666100
N	-12.74674500	-0.50851900	-0.76650100
H	-11.29234600	-1.71558200	0.14227200
H	-11.59081600	-0.17016100	0.94564800
C	-13.83693600	-1.13920300	0.07400100
H	-14.77832600	-1.03075900	-0.49014600
H	-13.87863200	-0.61421500	1.03002000
H	-13.58324500	-2.18905100	0.23120400
C	-13.11498500	0.93822900	-0.99711600
H	-14.11127100	0.94667500	-1.46430400
H	-12.37587100	1.39178300	-1.65656800
H	-13.12240300	1.44834400	-0.03180700
C	-12.71929300	-1.21977000	-2.09978200
H	-12.45024600	-2.26338400	-1.92560300
H	-11.98145600	-0.74106500	-2.74262500
Br	-16.09256000	-0.32373500	-2.42985900
H	-13.73056700	-1.12986000	-2.52736600
C	-0.63600400	9.86736100	1.69546600
C	-0.13389500	9.22422600	0.35148900
C	-0.83312300	11.27836800	-0.20392500
C	-1.06770700	11.33163300	1.28770500
H	-1.44464800	9.28341800	2.13381100
H	0.18179400	9.87612000	2.42291300
H	-2.11249800	11.53563400	1.53518300
H	-0.45480500	12.07850300	1.80061400
C	0.47532600	10.52109700	-0.41177800
C	-1.89120000	10.46855000	-0.88538900
H	-1.91708200	10.53606500	-1.97826200
H	-2.90644500	10.63260300	-0.51305100
C	0.85616600	10.23792300	-1.87584300
H	1.05952200	11.18100800	-2.39344100
H	1.76635500	9.63107600	-1.92626600
H	0.08435000	9.70967300	-2.44093800
C	1.70529100	11.11921200	0.29089300
H	2.57573400	10.46438700	0.17868100
H	1.95510500	12.07942400	-0.17238300
H	1.55908200	11.29679400	1.35711300
C	-1.39514800	9.01635700	-0.50305000
O	-1.89108500	7.98030400	-0.86261400
C	0.81165600	8.03948800	0.47018400
H	1.09352700	7.64849000	-0.50952300
H	1.71882800	8.33285900	1.00479900
S	0.26338000	6.56446400	1.41900800
O	-0.86849900	6.90539700	2.28548500
O	1.46829600	5.98234700	2.02586200
O	-0.21342600	5.62529700	0.21480900
H	-0.13681100	4.61879900	0.46407400
C	7.18766500	-4.02837600	5.33801800
C	6.51181300	-4.44291200	3.98407200
C	8.01710100	-6.05305100	4.39573500
C	8.20440200	-5.19923300	5.63013200
H	7.66076400	-3.05310000	5.25968200
H	6.42803200	-3.95184500	6.11697600
H	9.22990200	-4.83444900	5.72606100
H	7.94913000	-5.71313600	6.56009200

C	6.51984900	-6.05819800	4.10437600
C	8.65789100	-5.40933800	3.20597500
H	8.73727100	-6.03467000	2.31158600
H	9.64076500	-4.97004100	3.39500200
C	6.09977700	-6.76631900	2.80554600
H	6.32815200	-7.83262500	2.87479400
H	5.02069800	-6.67049700	2.64784200
H	6.59223000	-6.38085400	1.91164700
C	5.63205800	-6.58912400	5.23824600
H	4.57211600	-6.45772500	4.99732500
H	5.81114000	-7.66025800	5.36162100
H	5.81554500	-6.11087800	6.20130900

C	7.60295800	-4.27526000	2.91519600
O	7.64231900	-3.48658300	2.00878300
C	5.15735800	-3.81985800	3.68894900
H	4.75780400	-4.15084300	2.72757700
H	4.44192600	-4.07820700	4.47353900
S	5.03352000	-1.99003100	3.63540800
O	6.24041400	-1.34509400	4.17712500
O	3.73602100	-1.62917500	4.22146300
O	4.97745500	-1.77241700	2.05591300
H	4.91147200	-0.75401400	1.88309000
H	-0.77558021	12.26274959	-0.61935456
H	8.38621244	-7.04573678	4.54815447

Coordinates of optimized geometry: **TANI(BinPO$_4$H)$_2$-PTAB** E = -7025.45579063 a.u.

	X	Y	Z
C	3.10051700	2.71631400	-0.61435700
H	3.03819700	3.80161000	-0.65146600
C	4.30180200	2.09719500	-0.70437200
H	5.20165200	2.69402900	-0.79079900
C	4.42021900	0.64852800	-0.61671600
C	3.19390400	-0.10082300	-0.38002700
H	3.25502700	-1.18605500	-0.33765400
C	1.99138100	0.51678500	-0.29258500
H	1.09234600	-0.07936400	-0.19409200
C	1.87124500	1.96533800	-0.38953500
N	5.54051300	-0.03921400	-0.71316800
N	0.75010000	2.64897500	-0.30308600
C	6.75620100	0.48300100	-1.15250900
C	9.31795400	1.35837100	-2.01420100
C	7.93971400	-0.04579300	-0.59204000
C	6.88930000	1.43304200	-2.19113800
C	8.13860500	1.85126300	-2.61439500
C	9.19029400	0.39902200	-0.98701700
H	7.85839000	-0.79258100	0.19023600
H	6.00759600	1.79538800	-2.70791600
H	8.21598500	2.55473700	-3.43953300
H	10.07240600	0.01564400	-0.48958700
C	-0.46995500	2.10413100	0.12037500
C	-3.00821300	1.18721000	0.95305700
C	-0.59278100	1.21463900	1.20707100
C	-1.64983900	2.55817600	-0.50052100
C	-2.90131000	2.09738900	-0.11244100
C	-1.84177800	0.76815200	1.61374400
H	0.28891300	0.90892300	1.75931000
H	-1.57197000	3.27900700	-1.30711800
H	-3.79611600	2.44056000	-0.61118800
H	-1.91440800	0.09396600	2.46433400
N	-4.23534800	0.67527100	1.41961700
N	10.53599200	1.85576600	-2.44938400
H	-4.15865100	0.03314400	2.19646000
H	10.48708600	2.72785700	-2.95577700
C	11.83482300	1.35810400	-2.24440500
C	14.48244400	0.46199800	-1.92884800
C	12.90023900	2.27338100	-2.20461700
C	12.11212400	-0.01516600	-2.14165900
C	13.42636600	-0.44958300	-1.97374100
C	14.21026300	1.82599000	-2.05577100
H	12.69192800	3.33766700	-2.28176600
H	11.30878700	-0.73841200	-2.22020000
H	13.62393400	-1.51479200	-1.89461300
H	15.02004200	2.54933300	-2.02793600
H	15.50302900	0.11485800	-1.80253600
C	-5.51454100	0.94883900	0.98847100
O	-5.77920500	1.70767200	0.06554600
C	-6.59862900	0.22268100	1.79380000
H	-6.58078800	0.62428700	2.81586900
H	-6.33986700	-0.84168300	1.87958600
C	-7.99827300	0.39338700	1.19370500
H	-8.72902100	0.06591200	1.94288600
H	-8.16981400	1.46098100	1.01724800
C	-8.19342900	-0.37352600	-0.12356900
H	-8.07516800	-1.45218500	0.05340900
H	-7.40049600	-0.07715000	-0.81581400
C	-9.54197600	-0.11198600	-0.82288800
H	-9.52158700	-0.59853100	-1.80303200
H	-9.64481600	0.96391400	-0.99983900
C	-10.72134900	-0.63532900	-0.00305700
N	-12.09512700	-0.50777100	-0.63463000
H	-10.58976000	-1.70145700	0.20835600
H	-10.79041100	-0.11165200	0.95384100
C	-13.12024400	-1.05335100	0.33594500
H	-14.10739100	-0.95335500	-0.14251900
H	-13.06320900	-0.47053100	1.25703200
H	-12.87864500	-2.09863200	0.53695600
C	-12.44812900	0.93330900	-0.92401200
H	-13.48119600	0.93628700	-1.30650300
H	-11.75792200	1.32396000	-1.67082600
H	-12.36050400	1.50006500	0.00497100
C	-12.20161300	-1.30113100	-1.91665700
H	-11.93948800	-2.33864100	-1.69997200
H	-11.51433600	-0.88310600	-2.65119300
Br	-15.54257800	-0.27269800	-2.05165100
H	-13.24449600	-1.20968800	-2.26070200
C	1.05363900	8.75876200	-1.13028100
C	0.57266300	9.24434200	-2.33692900
C	1.06314200	10.52167100	-2.78635000
C	1.92469200	11.28679300	-1.93015600
C	2.31266300	10.75436800	-0.67199600
C	1.90506000	9.50225800	-0.28613900
H	0.15321700	10.46946000	-4.75308100
C	0.76716800	11.04907700	-4.07451200
C	2.40010800	12.55215800	-2.36884000
H	2.96203400	11.34220400	-0.02918800
H	2.21648000	9.05796500	0.65257900
C	2.07247400	13.04158900	-3.61181100
C	1.25768100	12.27231100	-4.47500400
H	3.04261800	13.12225300	-1.70262100
H	2.44728700	14.00742300	-3.93759700
H	1.02073200	12.64688900	-5.46667000
C	-0.84730500	6.27814500	-4.25384600
C	-2.03683600	6.75943700	-4.73956400
C	-2.48437400	8.06256900	-4.39560000
C	-1.65962100	8.89735500	-3.56881500
C	-0.37323700	8.41549700	-3.13592800
C	-0.03936600	7.10517600	-3.44484100
H	-4.35403000	7.90101400	-5.46751900
H	-0.49863800	5.27542400	-4.47496500
H	-2.66379600	6.13716100	-5.37222600
C	-3.74837800	8.54271600	-4.83265600
C	-2.17933300	10.16184700	-3.17342500
C	-3.41645000	10.59045600	-3.60147500
C	-4.20744600	9.78175100	-4.45068100
H	-1.59276100	10.79048700	-2.51445400
H	-3.79079500	11.55747700	-3.27806700
H	-5.17813900	10.13484300	-4.78608800
O	0.66109400	7.49834800	-0.68420200
O	1.15956900	6.56249100	-2.99034800
P	1.31917600	6.19285600	-1.40727300
O	0.23537100	5.10493700	-1.07473400
H	0.57185600	4.18165800	-0.75806400
O	2.73089200	5.88831600	-1.08284300
C	5.06355500	-5.96214700	1.13255300

C	5.68664800	-6.85188700	0.27049500
C	5.21031700	-8.21107300	0.25429100
C	4.21012400	-8.61879100	1.19982900
C	3.67623500	-7.66591100	2.10760300
C	4.07605300	-6.35423900	2.06062900
H	6.37726000	-8.87294700	-1.44874100
C	5.65587500	-9.17071200	-0.69749400
C	3.74773800	-9.96247700	1.19140300
H	2.92308100	-7.98459100	2.82293700
H	3.65750600	-5.59980400	2.71728800
C	4.22027600	-10.86992200	0.27207700
C	5.17444200	-10.46124700	-0.68876000
H	2.99831700	-10.25584300	1.92211600
H	3.85397600	-11.89229500	0.27177100
H	5.52662200	-11.17042200	-1.43230900
C	7.48537000	-4.78319100	-2.37070800
C	8.70870800	-5.39646900	-2.46874200
C	9.04725000	-6.47730900	-1.61185900
C	8.08070300	-6.95767300	-0.66545100
C	6.76866100	-6.36438900	-0.63057800
C	6.53730000	-5.26424200	-1.44284700
H	11.05137100	-6.70445300	-2.38793200
H	7.21522400	-3.93507200	-2.98999900
H	9.44359100	-5.04400700	-3.18735800
C	10.33684800	-7.07252000	-1.65598600
C	8.48501700	-7.98253900	0.23528700
C	9.74904900	-8.52677300	0.17671300
C	10.68396600	-8.07984900	-0.78609500
H	7.78561400	-8.32978100	0.98604400
H	10.03218200	-9.30323400	0.88151200
H	11.67473100	-8.52280300	-0.82639900
O	5.43805300	-4.61968500	1.13644500
O	5.31002500	-4.60928000	-1.40063200
P	4.93871600	-3.68684800	-0.10408400
O	6.00141400	-2.53208800	-0.01444700
H	5.69217100	-1.58757700	-0.29553300
O	3.50716400	-3.31206900	-0.13387100

3. Attempts to determine the CAC value of TANI-PTAB and $TANI(TFA)_2$-PTAB

The CAC value of EB-state **TANI-PTAB** was determined in earlier studies (*J. Am. Chem. Soc.* 2015, 137, 14288) to be 0.1 mM. We furthermore attempted to obtain the CAC value of **$TANI(TFA)_2$-PTAB** in aqueous solution using the ratio of peak intensities at 373 nm and 383 nm (I_{373}/I_{383}) from pyrene excitation spectra. However, we failed to obtain a reliable CAC value as the degree of acid doping depends on the concentration, which can be seen in the "Dilution de-doping self-assembly" section discussed in the SI, page S21.

4. Scattering analysis of 4 mM $TANI(TFA)_2$-PTAB

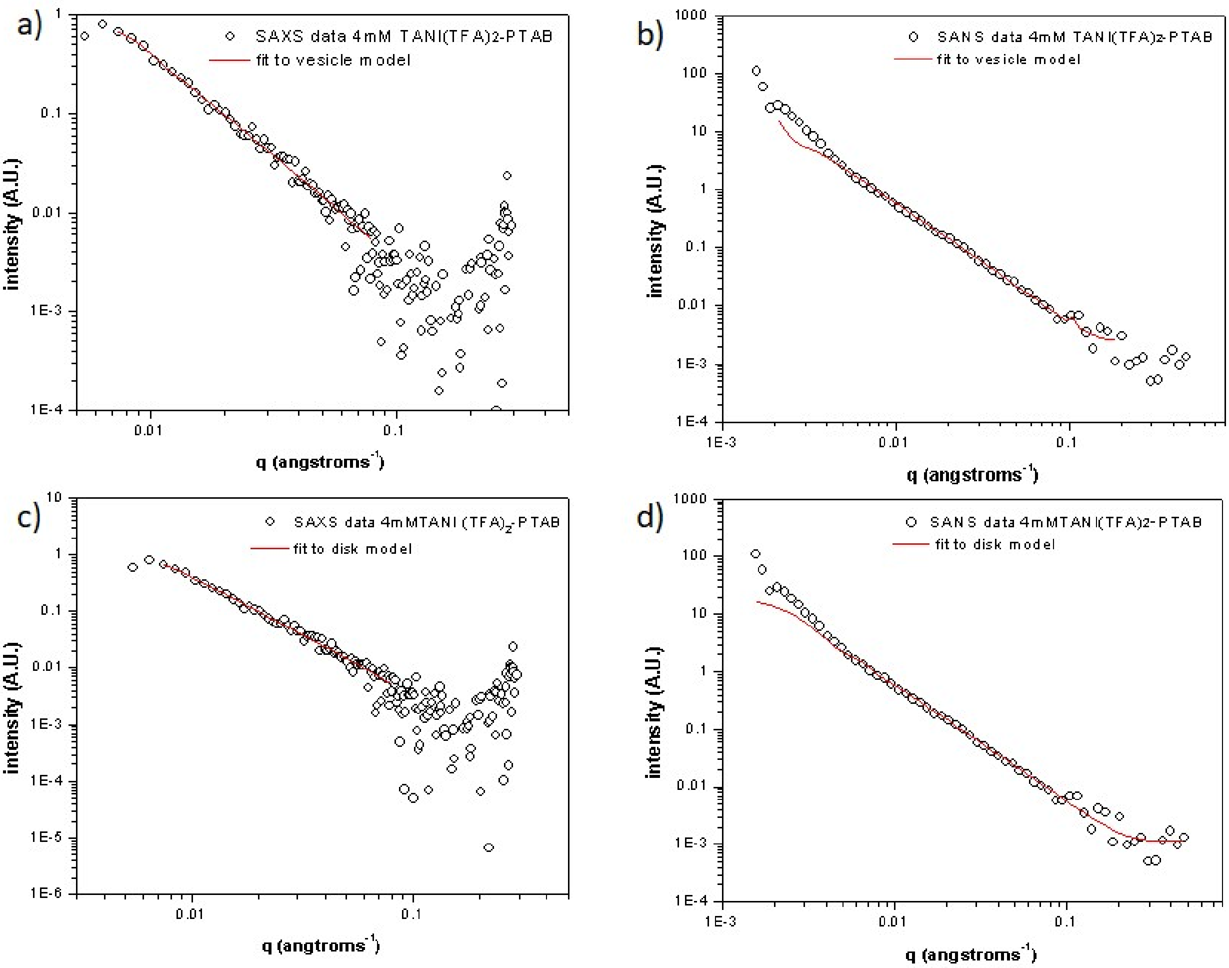

Figure S4. (a) Solution SAXS data for 4 mM ES **$TANI(TFA)_2$-PTAB** in H_2O (open circles) with a fit to a vesicle model overlaid (solid red line); (b) Solution SANS data for 4 mM ES **$TANI(TFA)_2$-PTAB** in D_2O (filled circles) with a fit to the vesicle model overlaid; (c) Solution SAXS data for 4 mM ES **$TANI(TFA)_2$-PTAB** in H_2O (open circles) with a fit to a disk model overlaid (solid red line); (d) Solution SANS data for 4 mM ES **$TANI(TFA)_2$-PTAB** in D_2O (filled circles) with a fit to the disk model overlaid.

Both SAXS and SANS data from 4mM TANI doped with TFA show a ~Q^{-2} dependence, consistent with the presence of *either* disk-like objects *or* a unilamellar structure. Fitting both the SAXS (Figure S4a) and SANS data (Figure S4b) to a

vesicle model (Table S2 and S3) gives a size far exceeding that seen by AFM, TEM or DLS though the fit is poor at low Q values. However, the thickness of ~3 nm is commensurate with the thickness of one interdigitated bilayer of **TANI(TFA)$_2$-PTAB.** Fitting both SAXS and SANS data to a disk model (Table S4 and S5) gives greater radii than observed by other techniques and a thickness of ~2 nm. Note that a high polydispersity has been necessary to fit the data.

Table S2. Best fit parameters from fitting a vesicle model from SAXS data (σ is one standard deviation)

Fit parameter	Value	Error
Scale	27.2×10^{-6}	1.3×10^{-6}
Background (cm^{-1})	0.002	0.004
SLD of solvent (cm^{-2})	1.25×10^{-5}	-
SLD of solute (cm^{-2})	1.32×10^{-5}	-
Radius (nm)	40.1	5.1
Thickness (nm)	2.9	0.1
Polydispersity ratio (σ/radius)	0.4	0.009
X^2/N_{points}	1.37	

Table S3. Best fit parameters from fitting a vesicle model from SANS data (σ is one standard deviation)

Fit parameter	Value	Error
Scale	0.0025	0.0005
Background (cm^{-1})	0.00013	1.7×10^{-5}
SLD of solvent (cm^{-2})	6.36×10^{-6}	-
SLD of solute (cm^{-2})	1.5×10^{-6}	-
Radius (nm)	78.4	0.41
Thickness (nm)	2.9	0.4
Polydispersity ratio (σ/radius)	0.44	0.0096
X^2/N_{points}	9.11	

Table S4. Best fit parameters from fitting a disk model from SAXS data (σ is one standard deviation)

Fit parameter	Value	Error
Scale	0.0063	7.6×10^{-5}
Background (cm^{-1})	0.001	0.0005
SLD of solvent (cm^{-2})	1.25×10^{-5}	-
SLD of solute (cm^{-2})	1.32×10^{-5}	-
Radius (nm)	62.1	2.7
Length (nm)	2.0	0.03
Polydispersity ratio (σ/radius)	-	-
X^2/N_{points}	1.06	

Table S5. Best fit parameters from fitting a disk model from SANS data (σ is one standard deviation)

Fit parameter	Value	Error
Scale	0.0002	0.0009
Background (cm^{-1})	0.0011	$3.7x10^{-5}$
SLD of solvent (cm^{-2})	6.36×10^{-6}	-
SLD of solute (cm^{-2})	1.5×10^{-6}	-
Radius (nm)	87.7	2.5
Length (nm)	1.8	0.3
Polydispersity ratio (σ/radius)	-	-
X^2/N_{points}	6.05	

5. Dilution de-doping self-assembly

We investigated the effect of dilution on doped states (and thus also formed structures). Dilution of **TANI(TFA)$_2$-PTAB** from 1 mM to 0.01 mM led to de-doping, indicated by corresponding changes in the UV-Vis spectra, shown in Figure S5a. De-doping is shown by the dilution-induced appearance of absorbance bands assigned to the HOMO-LUMO transition of EB **TANI-PTAB** (λ_{max} = 578 nm), [7] the onset of which was at 0.165 mM. The absence of bands at 780 nm typical for ES state in 0.067 mM sample suggested that, below this concentration, **TANI(TFA)$_2$-PTAB** was fully de-doped. As shown in Figure S5b, the pH increased from 3.0 (1 mM) to 5.2 (0.01 mM) during the whole dilution process, and the pH for de-doping was found around 3.8 in 0.067 mM sample. These results show that ES **TANI(TFA)$_2$-PTAB** can be de-doped to form EB **TANI-PTAB** with dilution by changing pH.

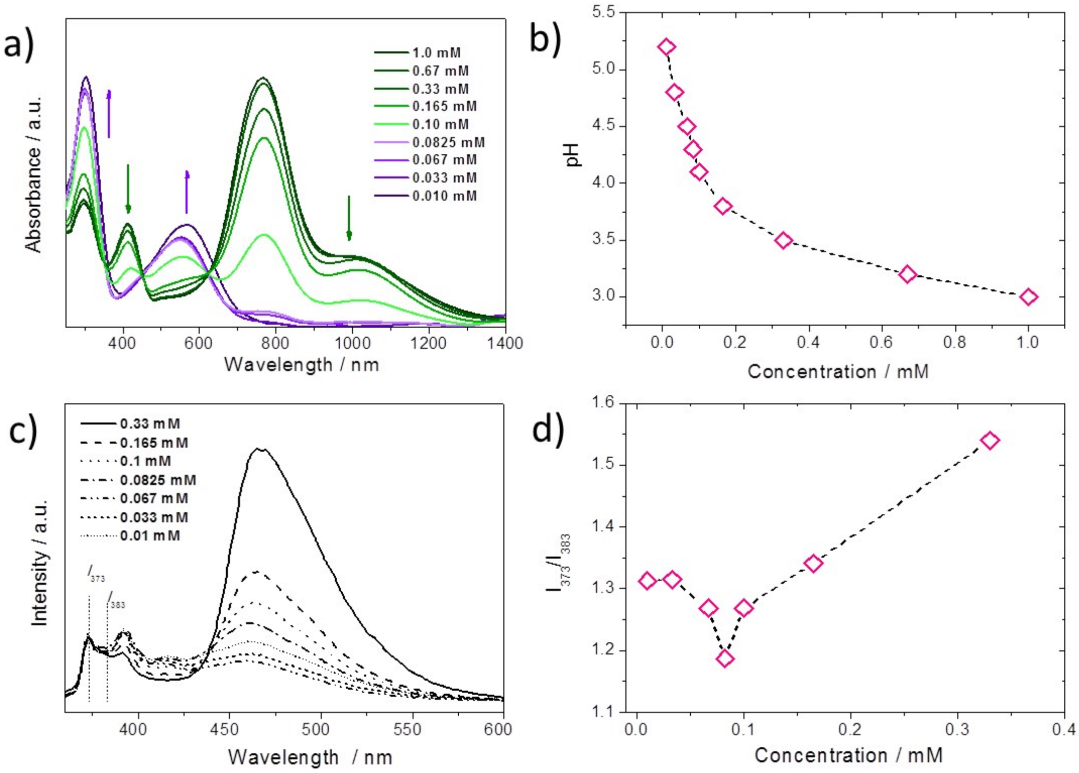

Figure S5. (a) UV-Vis-NIR spectra showing the transition from doped ES **TANI(TFA)$_2$-PTAB** to dedoped EB **TANI-PTAB** on dilution from 1 mM to 0.1 mM and (b) the corresponding pH value upon dilution. (c) Normalized fluorescence emission spectra for pyrene in the presence of varying concentrations of **TANI(TFA)$_2$-PTAB** and (d) the corresponding I_{373}/I_{383} ratio.

Pyrene can act as a fluorescent probe to enable highly sensitive measurements of local polarity, by determination of the intensity ratio of peak $\mathrm{I}/$ peak III (I_{373}/I_{383}) of pyrene's emission spectrum.[8, 9] Generally, a value of I_{373}/I_{383} above 1.5 indicates that pyrene experiences a hydrophilic environment, whereas values as low as 0.6 can be observed in hydrophobic environments.[10, 11] In Figure S5d, the value of 1.54 for 0.33 mM ES **TANI(TFA)$_2$-PTAB** suggests a hydrophilic environment, which is in agreement with pyrene being contained within the proposed bilayer vesicular structures. The decrease in value of I_{373}/I_{383} to 1.18 upon dilution from 0.33 mM to 0.0825 mM indicates a change in the local environment of pyrene, suggesting a possible structural transformation occurs.

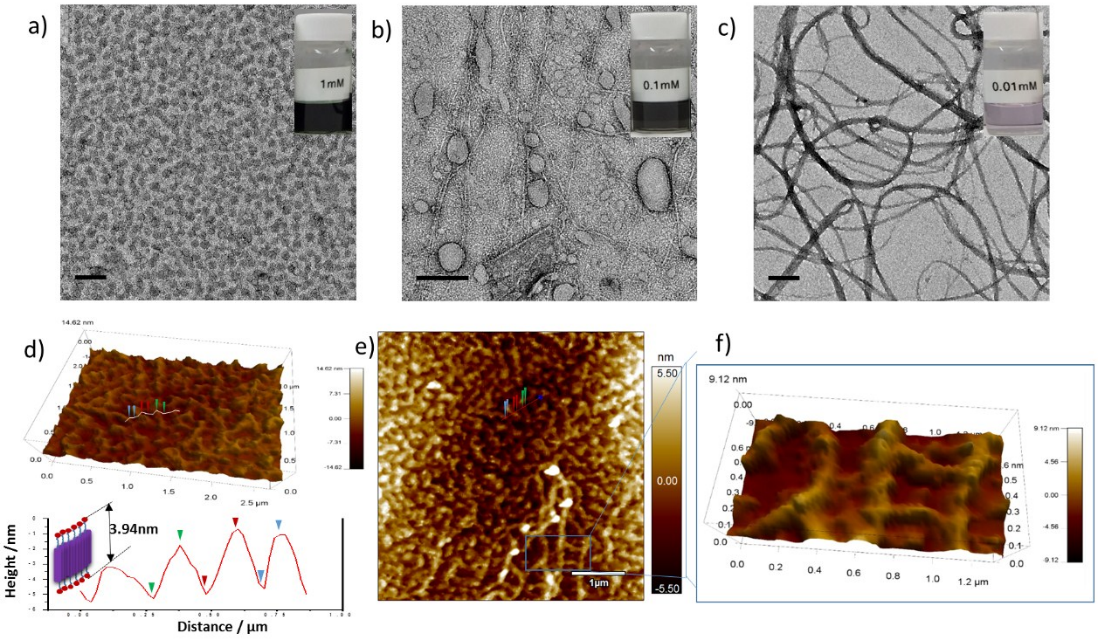

Figure S6. (a-c) TEM images (stained with 1% uranyl acetate) of (a) 1 mM, (b) 0.1 mM and (c) 0.01 mM **TANI(TFA)$_2$-PTAB** samples, with photographs of the corresponding solutions inset. (e) AFM height image of **TANI(TFA)$_2$-PTAB (**2 mM) on HOPG, that was rinsed with 100 µl deionised water after deposition, and the corresponding (d) cross-sectional height profile and (f) 3D image. Scale bars: 200 nm.

This structural transformation was further investigated by TEM and AFM. From the TEM images of samples with different concentration upon dilution in Figure S6, we can see that the vesicles formed by the ES state (1 mM, Figure S6a) start to merge into to larger flexible bilayer structures to incorporate the mixture of ES and EB states (0.1 mM, Figure S6b), followed by re-formation of nanowires in the EB state (0.01 mM, Figure S6c). Moreover, a similar transition process can be observed from AFM, where an ES-state vesicular sample was imaged after rinsing with water, to simulate dilution-induced transition to the EB state. As shown in Figure S6f, a few vesicles remained in the sample, but the dominant species present were nanowires with an average height of about 3.7 nm, which is consistent with the thickness of two stacked **TANI-PTAB** molecules (Figure S6d).

These results are clearly indicative of dilution-induced dedoping **TANI(TFA)$_2$-PTAB**, with an associated switch in self-assembly behaviour in aqueous solution.

6. Supplementary analysis

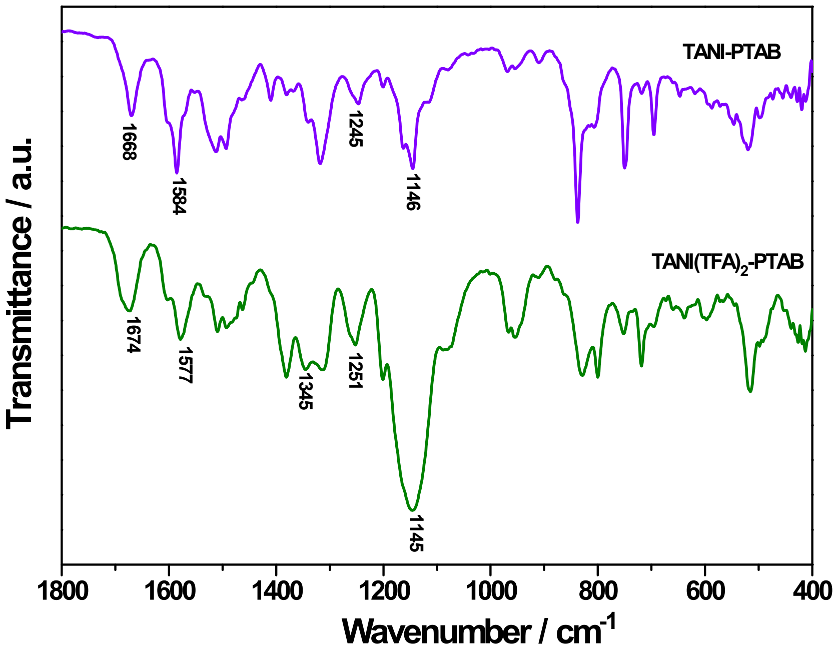

Figure S7. FT-IR spectra of **TANI-PTAB** and 2 mM **TANI(TFA)$_2$-PTAB** recorded in the solid state.

The characteristic stretching vibrations of the **TANI-PTAB** sample are located at 1668 cm^{-1} ($\nu_{C=C}$ for quinoid rings), 1584 cm^{-1} ($\nu_{C=C}$ for benzenoid rings), 1245 cm^{-1} (ν_{C-N}) and 1146 cm^{-1} (γ_{C-H} out-of-plane of 1,4-aromatic substituted benzene rings), which are typical for TANI in EB state. The characteristic bands of **TANI(TFA)$_2$-PTAB** are located at 1674 cm^{-1} ($\nu_{C=C}$ for quinoid rings), 1577 cm^{-1} ($\nu_{C=C}$ for benzenoid rings), 1251 cm^{-1} ($\nu_{C-N^{+\cdot}}$) and 1145 cm^{-1} ($\nu_{-NH^{+}=}$), which are typical for TANI in the ES state. Moreover, no obvious negative bands (Evans holes) cutting the broad infrared adsorption centered near 1100 cm^{-1}, which correspond to asymmetric interchain $NH^{+}\cdots N$ hydrogen bonds [12, 13], were observed in the FT-IR spectrum of the **TANI(TFA)$_2$-PTAB** sample.

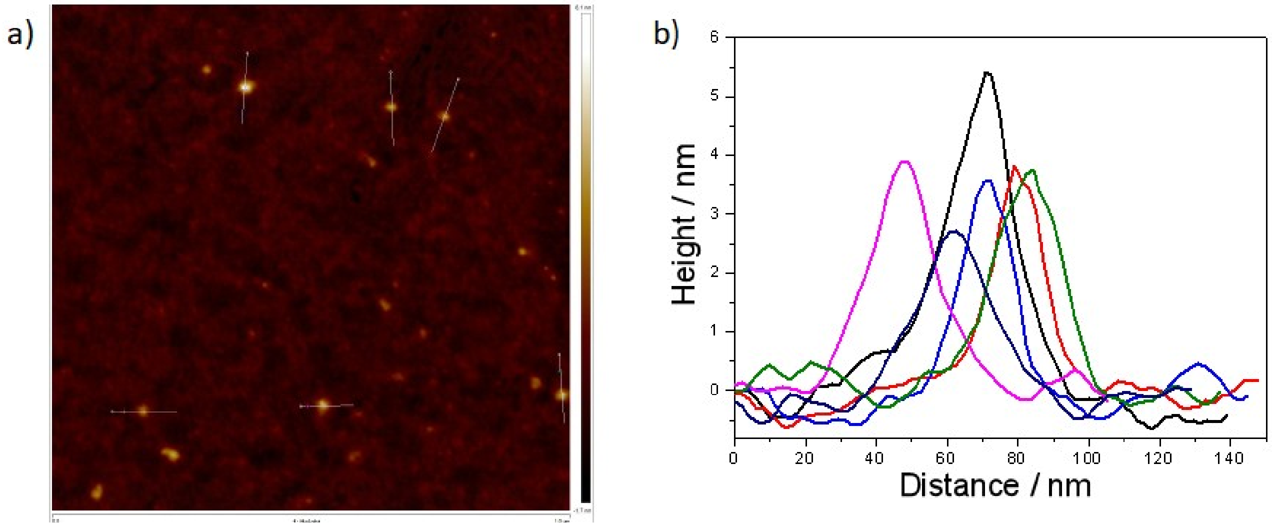

Figure S8. AFM image of 2 mM **TANI(TFA)$_2$-PTAB** sample on mica surface with particles embedded in a film (a) and the corresponding cross-sectional height profile of those protruding most prominently from the film.

These particles were surrounded and possible even covered in additional material that has no specific structure which may be introduced in the process of drop casting as sample structure is dependent on concentration and further washes are not possible. The average height was measured around 3.5 ± 0.6 nm for the particles with average diameter of 30 nm which is lower than that measured for isolated particles on mica. Although not useful for accurate measurement of particle dimensions, the presence of the particles embedded in the film demonstrates that those observed individually on the mica surface without contaminant are not the result of the drying mechanism.

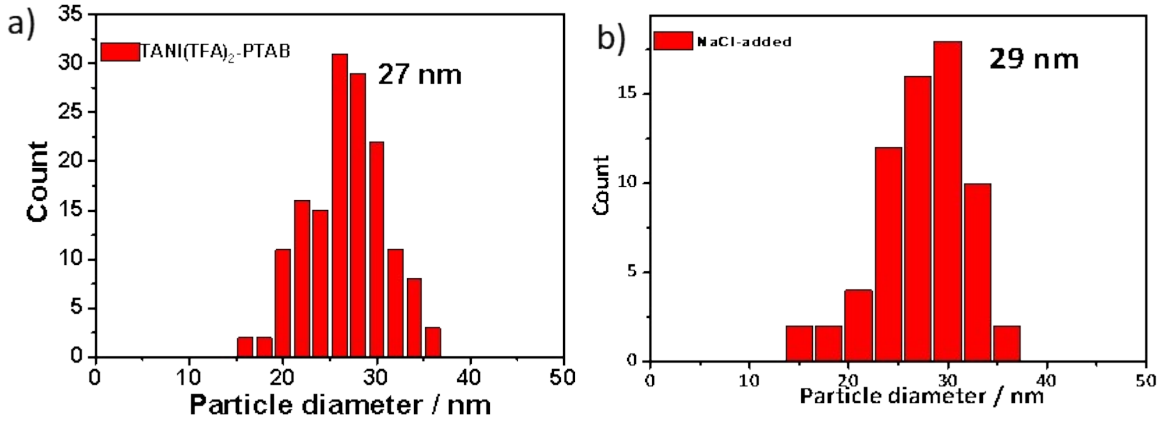

Figure S9. (a) Particle size distribution for 4 mM **TANI(TFA)$_2$-PTAB** sample counted from TEM image; (b)Particle size distribution for NaCl-added 1mM **TANI(TFA)$_2$-PTAB** sample counted from TEM image.

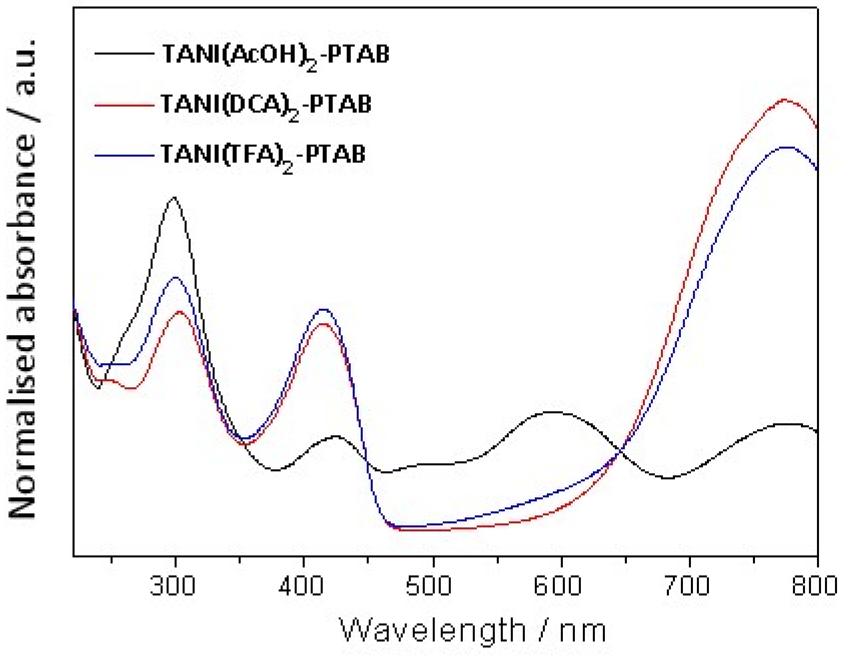

Figure S10. UV-Vis spectra of **TANI(AcOH)$_2$-PTAB, TANI(DCA)$_2$-PTAB**, and **TANI(TFA)$_2$-PTAB** samples.

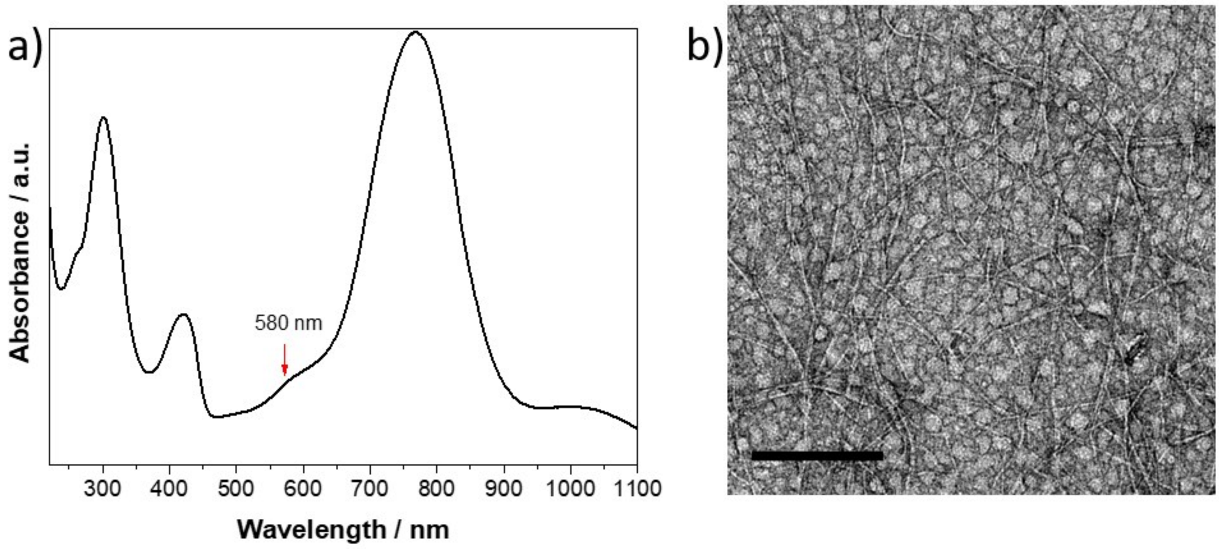

Figure S11. (a) UV-Vis-NIR spectrum of **AcOH**-doped **TANI-PTAB** with a mole ratio of 4:1 (**AcOH:TANI-PTAB**) left to age for 24 h, and (b) TEM image of this sample showing the co-existence of both fibers and vesicles.

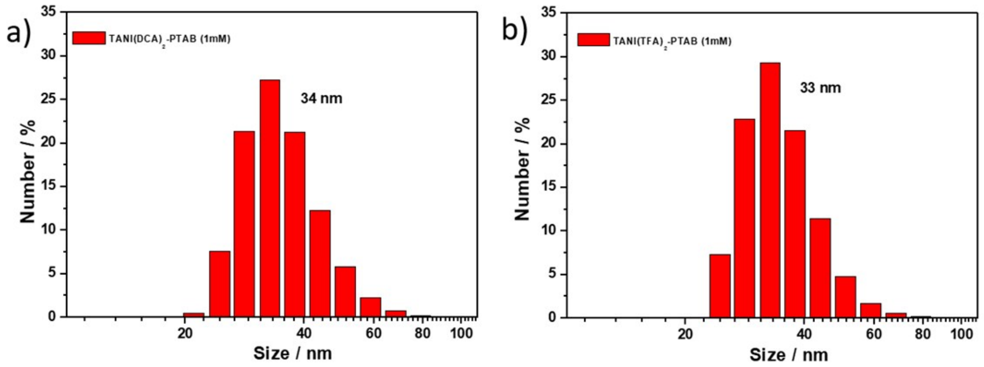

Figure S12. Number distributions of DLS particles sizes for 1 mM **TANI-(DCA)$_2$-PTAB** (a) and **TANI-(TFA)$_2$-PTAB** (b) solution samples.

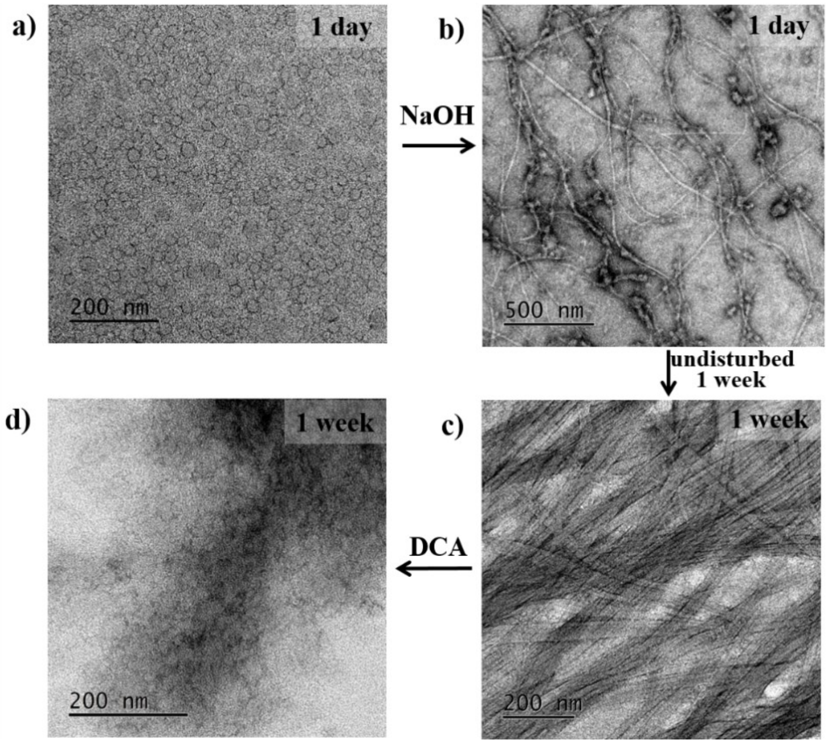

Figure S13. TEM images (stained with 1% uranyl acetate) of **TANI(DCA)$_2$-PTAB** (1 mM) after standing 1 day, (b) NaOH de-doped-**TANI-PTAB** 1 day, (c) the same sample as (b) after 1 week, and (d) **TANI(DCA)$_2$-PTAB** (0.92 mM) obtained by treating the same sample as (b, c) with **DCA**, after standing 1 week.

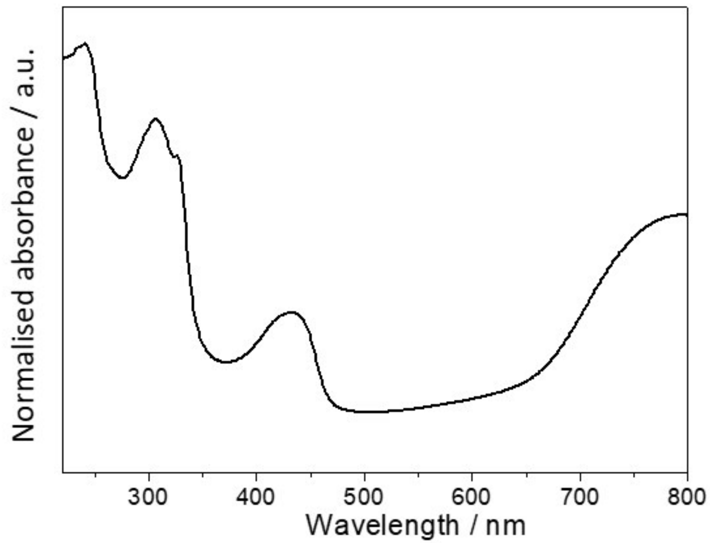

Figure S14. UV-Vis spectra of **TANI(BINPO$_4$H)$_2$-PTAB** samples.

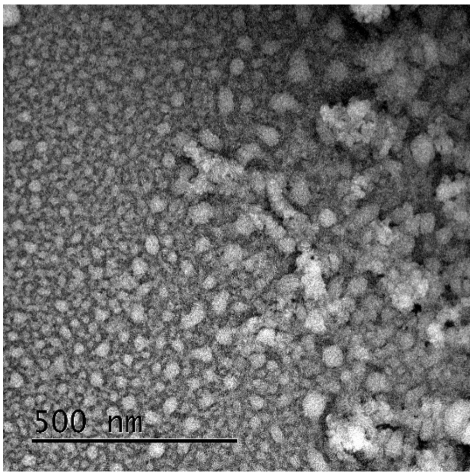

Figure S15. TEM image of **TANI(BINPO$_4$H)$_2$-PTAB** samples.

Reference

[1] X.P. Chen, J.K. Jiang, Q.H. Liang, N. Yang, H.Y. Ye, M. Cai, L. Shen, D.G. Yang, T.L. Ren, First-principles study of the effect of functional groups on polyaniline backbone, *Scientific Reports*, 5 (**2015**) 16907.

[2] A.D. Becke, Density‐functional thermochemistry. III. The role of exact exchange, *The Journal of Chemical Physics*, 98 (**1993**) 5648-5652.

[3] C. Lee, W. Yang, R.G. Parr, Development of the Colle-Salvetti correlation-energy formula into a functional of the electron density, *Physical Review B*, 37 (**1988**) 785-789.

[4] S.H. Vosko, L. Wilk, M. Nusair, Accurate spin-dependent electron liquid correlation energies for local spin density calculations: a critical analysis, *Canadian Journal of Physics*, 58 (**1980**) 1200-1211.

[5] P.J. Stephens, F.J. Devlin, C.F. Chabalowski, M.J. Frisch, Ab Initio Calculation of Vibrational Absorption and Circular Dichroism Spectra Using Density Functional Force Fields, *The Journal of Physical Chemistry*, 98 (**1994**) 11623-11627.

[6] M.J.T.R. Frisch, G. W.; Schlegel, H. B.; Scuseria, G. E.; Robb, M. A.; Cheeseman, J. R.; Scalmani, G.; Barone,, B.P. V.; Mennucci, G. A.; Nakatsuji, H.; Caricato, M.; Li, X.; Hratchian, H. P.; Izmaylov, A. F.; Bloino, J.;, G.S. Zheng, J. L.; Hada, M.; Ehara, M.; Toyota, K.; Fukuda, R.; Hasegawa, J.; Ishida, M.; Nakajima, T.;, Y.K. Honda, O.; Nakai, H.; Vreven, T.; Montgomery, J. A. J.; Peralta, J. E.; Ogliaro, F.; Bearpark, M.; Heyd, J. J.;, E.K. Brothers, K. N.; Staroverov, V. N.; Kobayashi, R.; Normand, J.; Raghavachari, K.; Rendell, A.; Burant, J., S.S.T. C.; Iyengar, J.; Cossi, M.; Rega, N.; Millam, J. M.; Klene, M.; Knox, J. E.; Cross, J. B.; Bakken, V.;, C.J. Adamo, J.; Gomperts, R.; Stratmann, R. E.; Yazyev, O.; Austin, A. J.; Cammi, R.; Pomelli, C.;, J.W.M. Ochterski, R. L.; Morokuma, K.; Zakrzewski, V. G.; Voth, G. A.; Salvador, P.; Dannenberg, J. J.;, S.D. Dapprich, A. D.; Farkas, Ö.; Foresman, J. B.; Ortiz, J. V.; Cioslowski, J.; Fox, D. J. Gaussian 09, **2009**, W. CT.

[7] Y. Wang, J. Liu, H.D. Tran, M. Mecklenburg, X.N. Guan, A.Z. Stieg, B.C. Regan, D.C. Martin, R.B. Kaner, Morphological and dimensional control via hierarchical assembly of doped oligoaniline single crystals[J], *Journal of the American Chemical Society*, 134 (**2012**) 9251-9262.

[8] C. Schatz, E.G. Smith, S.P. Armes, E.J. Wanless, Reversible pH-triggered encapsulation and release of pyrene by adsorbed block copolymer micelles, *Langmuir,* 24 (**2008**) 8325-8331.

[9] K. Kaushlendra, S. Asha, Microstructural Reorganization and Cargo Release in Pyrene Urethane Methacrylate Random Copolymer Hollow Capsules, *Langmuir,* 28 (**2012**) 12731-12743.

[10] F.M. Winnik, Photophysics of preassociated pyrenes in aqueous polymer solutions and in other organized media, *Chemical Reviews,* 93 (**1993**) 587-614.

[11] K. Kalyanasundaram, J.K. Thomas, Environmental effects on vibronic band intensities in pyrene monomer fluorescence and their application in studies of micellar systems, *Journal of the American Chemical Society*, 99 (**1977**) 2039-2044.

[12] P. Colomban, A. Gruger, A. Novak, A. Régis, Infrared and Raman study of polyaniline Part I. Hydrogen bonding and electronic mobility in emeraldine salts*, Journal of Molecular Structure*, 317 (**1994**) 261-271.

[13] W. Lyu, J. Feng, W. Yan, C.F.J. Faul, Self-assembly of tetra(aniline) nanowires in acidic aqueous media with ultrasonic irradiation, *Journal of Materials Chemistry C*, 3 (**2015**) 11945-11952.
